# Supplementary material for: Analysis of economic and educational spillover effects in PEPFAR countries
Source: PLoS One. 2023 Dec 29;18(12):e0289909. doi: 10.1371/journal.pone.0289909 (PMC10756543; doi:10.1371/journal.pone.0289909)
Supplement: S1 Appendix — Exhibit A. Cohorts of PEPFAR countries created for analysis. Exhibit B. Country list by groups. Exhibit C. Difference-in-difference (DID) methodology. Exhibit D. Missingness. Exhibit E. Sensitivity tests for PEPFAR impact on GDP growth per capita. Exhibit F. Sensitivity test for PEPFAR impact on female primary school disengagement. Exhibit G. Sensitivity test for PEPFAR impact on male primary school disengagement. Exhibit H. Sensitivity test for PEPFAR impact on female employment rates. Exhibit I. Sensitivity test for PEPFAR impact on male employment rates. (DOCX) [file pone.0289909.s001.docx]

**Analysis of Economic and Educational Spillover Effects in PEPFAR Countries**

**S1 Appendix Materials**

**Exhibit [A] – Exhibit [I]**

**Exhibit A. Cohorts of PEPFAR countries created for analysis**

- **Based on OGAC programmatic planning differences.** The PEPFAR program has provided strategic budget and programmatic guidance in several ways over the years. PEPFAR’s initial group of 15 focus countries were later replaced with a group of high-burden, highly supported countries that were required to submit and negotiate annual Country Operating Plans (COP). This intense annual planning process involves country officials and OGAC staff conducting performance reviews of prior programming, goal setting, and annual budget negotiations. Since 2004, 31 of the 90 PEPFAR countries have consistently been COP recipients. These countries are studied separately from all other PEPFAR recipients.

**Exhibit B. Country list by groups**

- **All PEPFAR-funded LMICs**

Afghanistan

Albania

Angola

Armenia

Bangladesh

Barbados

Belize

Benin

Bolivia

Botswana

Brazil

Burkina Faso

Burundi

Cambodia

Cameroon

Central African Republic

Chad

China

Comoros

Congo, Dem. Rep.

Congo, Rep.

Cote d'Ivoire

Djibouti

Dominican Republic

El Salvador

Eritrea

Estonia

Eswatini

Ethiopia

Gabon

Gambia, The

Georgia

Ghana

Guatemala

Guinea

Guinea-Bissau

Guyana

Haiti

Honduras

India

Indonesia

Jamaica

Jordan

Kazakhstan

Kenya

Kyrgyz Republic

Lao PDR

Lesotho

Liberia

Madagascar

Malawi

Mali

Mauritania

Mexico

Moldova

Mozambique

Myanmar

Namibia

Nepal

Nicaragua

Niger

Nigeria

North Macedonia

Pakistan

Papua New Guinea

Peru

Philippines

Romania

Russian Federation

Rwanda

Samoa

Sao Tome and Principe

Senegal

Seychelles

Sierra Leone

South Africa

Suriname

Tajikistan

Tanzania

Thailand

Timor-Leste

Togo

Trinidad and Tobago

Turkmenistan

Uganda

Ukraine

Uzbekistan

Vietnam

Zambia

Zimbabwe

- **COP-PEPFAR countries**

Angola

Botswana

Burundi

Cambodia

Cameroon

Congo, Dem. Rep.

Cote d'Ivoire

Dominican Republic

Eswatini

Ethiopia

Ghana

Guyana

Haiti

India

Indonesia

Kenya

Lesotho

Malawi

Mozambique

Myanmar

Namibia

Nigeria

Papua New Guinea

Rwanda

South Africa

Tanzania

Uganda

Ukraine

Vietnam

Zambia

Zimbabwe

- **Other PEPFAR-recipient countries**

Afghanistan

Albania

Armenia

Bangladesh

Barbados

Belize

Benin

Bolivia

Brazil

Burkina Faso

Central African Republic

Chad

China

Comoros

Congo, Rep.

Djibouti

El Salvador

Eritrea

Estonia

Gabon

Gambia, The

Georgia

Guatemala

Guinea

Guinea-Bissau

Honduras

Jamaica

Jordan

Kazakhstan

Kyrgyz Republic

Lao PDR

Liberia

Madagascar

Mali

Mauritania

Mexico

Moldova

Nepal

Nicaragua

Niger

North Macedonia

Pakistan

Peru

Philippines

Romania

Russian Federation

Samoa

Sao Tome and Principe

Senegal

Seychelles

Sierra Leone

Suriname

Tajikistan

Thailand

Timor-Leste

Togo

Trinidad and Tobago

Turkmenistan

Uzbekistan

- **Control group**

Algeria

American Samoa

Antigua and Barbuda

Argentina

Azerbaijan

Belarus

Bhutan

Bosnia and Herzegovina

Bulgaria

Cabo Verde

Chile

Colombia

Costa Rica

Croatia

Cuba

Czech Republic

Dominica

Ecuador

Egypt, Arab Rep.

Equatorial Guinea

Fiji

Grenada

Hungary

Iran, Islamic Rep.

Iraq

Kiribati

Korea, Dem. People’s Rep.

Kosovo

Latvia

Lebanon

Libya

Lithuania

Malaysia

Maldives

Marshall Islands

Mauritius

Mayotte

Micronesia, Fed. Sts.

Mongolia

Montenegro

Morocco

Nauru

Northern Mariana Islands

Oman

Palau

Panama

Paraguay

Poland

Serbia and Montenegro

Serbia

Slovak Republic

Solomon Islands

Somalia

Sri Lanka

St. Kitts and Nevis

St. Lucia

St. Vincent and the Grenadines

Syrian Arab Republic

Tonga

Tunisia

Turkey

Tuvalu

Uruguay

Vanuatu

Venezuela, RB

West Bank and Gaza

Yemen, Rep.

**Exhibit C. Difference-in-difference (DID) methodology**

The Difference in Difference method provides a direct estimate of the impacts of PEPFAR on outcomes, based on the counterfactual provided by a control group of countries. DID can be thought of as an extension of Quasi Experimental Design to account for unobserved variables potentially correlated with both an intervention and the outcomes that are assumed to remain fixed over time. This DID method has been widely used in the program evaluation literature to estimate treatment effects as a non-parametric alternative to parametric sample selection models (Wooldridge, 2002). The method can be used when pre and post data are available for countries that received PEPFAR funding and for those that did not (e.g., the control group). Characteristics of the control group countries were also measured in the baseline period. The first group of PEPFAR countries began receiving funding in late 2003, and the earliest health effects could first be measured in 2004, the beginning of our follow-up period. We also measure the outcomes for countries in the control group in the same post-2004 period. If we assume that countries may also have unobserved characteristics, λi, that are correlated with outcomes and that these characteristics remain fixed over time (e.g., unobserved health endowment), DID provides a method to control for these fixed, unobserved characteristics.

To see this, we defined the outcome equations for periods 1 and 2, as shown in equations 1a and 1b, respectively:

(1a) Y_i1_=B_0_ + B_1_X_i1_+B_2_λ_i_+є_i1_

(1b) Y_i2_=B_0_ + B_1_X_i2_+B_2_λ_i_+B_3_T_i_+є_i2_

Calculating the change in outcomes and explanatory variables between time 1 and time 2, and re-estimating the outcome equation, is equivalent to subtracting equation (1a) from (1b):

(2) (Y_i2_- Y_i1_)= (B_0_- B_0_)+ B_1_(X_i2_- X_i1_)+ B_2_ (λi- λ_i_) +B_3_T_i_+(є_i2_- є_i1_)

Which simplifies to:

(3) (Y_i2_- Y_i1_)= B_1_(X_i2_- X_i1_)+B_3_T_i_+(є_i2_- є_i1_)

In other words, the DID approach subtracts out unobserved fixed effects of countries that may be correlated with both treatment selection and outcomes.

Operationally, the DID model is easy to implement using three dummy variables in a panel data set: (1) a time dummy captures the overall differences in the mean value of the dependent variable between the baseline period (pre 2004) and the follow-up period; (2) A dummy variable for PEPFAR countries and 0 for control group countries; (3) an interaction dummy between the first two dummy variables, the coefficient on which estimates the impact of PEPFAR.

**Exhibit D. Missingness**

Missing baseline data (for the year 2004) were estimated using linear interpolation of existing data for all other years for most variables. Missing data on domestic private and government spending were calculated as a function of GDP per capita. HIV prevalence data were extracted from IHME if missing in the World Bank dataset.

**Exhibit E. Sensitivity tests for PEPFAR impact on GDP growth per capita**

- by PEPFAR/COP country income group
- by three five-year intervals of the post-treatment period for PEPFAR/COP country
- exclude China and India as PEPFAR/COP country
- use shorter years as pre-treatment period (1999-2003) for PEPFAR/COP country

**Exhibit [E]. DID results for PEPFAR impact on GDP growth per capita by PEPFAR country income group**

| **Variables** | **Low-income**  **PEPFAR countries** | | **Middle-income**  **PEPFAR countries** | | **All**  **PEPFAR countries** | |
| --- | --- | --- | --- | --- | --- | --- |
|  | **Unadjusted** | **Adjusted** | **Unadjusted** | **Adjusted** | **Unadjusted** | **Adjusted** |
| Time variable (=1 post-2004) | -0.287 | -0.129 | -0.287 | -0.112 | -0.287 | -0.112 |
|  | (0.352) | (0.364) | (0.358) | (0.368) | (0.333) | (0.343) |
| Intervention (=1 if PEPFAR) | -2.403*** | -2.292*** | -1.418*** | -1.152* | -1.977*** | -1.754*** |
|  | (0.382) | (0.592) | (0.418) | (0.509) | (0.317) | (0.389) |
| Interaction term (PEPFAR impact estimate) | 2.738*** | 2.663*** | 1.669** | 1.292* | 2.276*** | 2.072*** |
|  | (0.518) | (0.519) | (0.572) | (0.573) | (0.429) | (0.434) |
| Country income group (=1 if middle income) |  | n/a |  | n/a |  | 0.347 |
|  |  | n/a |  | n/a |  | (0.393) |
| BL population |  | 1.66e-09 |  | 4.26e-09*** |  | 2.98e-09*** |
|  |  | (1.19e-09) |  | (1.04e-09) |  | (7.38e-10) |
| BL per capita non-PEPFAR donor spending on health (constant $) |  | -0.00216 |  | -0.00628 |  | -0.00303 |
|  |  | (0.00511) |  | (0.00542) |  | (0.00473) |
| BL per capita domestic health spending (current $) |  | -0.00317** |  | -0.00292** |  | -0.00305*** |
|  |  | (0.00107) |  | (0.00105) |  | (0.000863) |
| BL GDP per capita, PPP (constant 2011 international $) |  | 0.000177*** |  | 0.000130*** |  | 0.000143*** |
|  |  | (0.0000392) |  | (0.0000358) |  | (0.0000322) |
| BL HIV prevalence (% of population ages 15-49) |  | -0.0500 |  | -0.00362 |  | 0.00213 |
|  |  | (0.0530) |  | (0.0539) |  | (0.0374) |
| BL life expectancy at birth |  | -0.0553 |  | -0.0481 |  | -0.00705 |
|  |  | (0.0413) |  | (0.0441) |  | (0.0319) |
| BL urban population (%) |  | -0.00841 |  | 0.00185 |  | -0.0129 |
|  |  | (0.00916) |  | (0.00940) |  | (0.00754) |
| BL school enrollment, secondary (% gross) |  | -0.0354*** |  | -0.0245* |  | -0.0300*** |
|  |  | (0.00973) |  | (0.0103) |  | (0.00802) |
| BL fertility rate (births per woman) |  | -0.961*** |  | -0.592** |  | -0.712*** |
|  |  | (0.195) |  | (0.212) |  | (0.151) |
| Recipient of US HIV aid before 2004 (=1 if yes) |  | 0.405 |  | -0.685 |  | 0.0136 |
|  |  | (0.356) |  | (0.461) |  | (0.270) |
| Constant | 2.875*** | 11.93*** | 2.875*** | 9.481** | 2.875*** | 7.691** |
|  | (0.262) | (3.384) | (0.266) | (3.519) | (0.247) | (2.515) |
| Observations | 3122 | 2858 | 2751 | 2453 | 4192 | 3865 |
| Adjusted R-squared | 0.017 | 0.033 | 0.004 | 0.021 | 0.015 | 0.035 |

Notes: Standard errors in parentheses; ***p < 0.001   **p < 0.01 * p< 0.05. Source: Our data came from four publicly available datasets: World Bank’s World Development Indicators; U.S. government’s foreignassistance.gov database; OECD Creditor Reporting System database; and the Institute of Health Metrics and Evaluation GBD Result’s Tool.

**Exhibit [E]. DID results for PEPFAR impact on GDP growth per capita by COP country income group**

| **Variables** | **Low-income**  **COP countries** | | **Middle-income**  **COP countries** | | **All**  **COP countries** | |
| --- | --- | --- | --- | --- | --- | --- |
|  | **Unadjusted** | **Adjusted** | **Unadjusted** | **Adjusted** | **Unadjusted** | **Adjusted** |
| Time variable (=1 post-2004) | -0.287 | -0.132 | -0.287 | -0.128 | -0.287 | -0.125 |
|  | (0.372) | (0.383) | (0.388) | (0.403) | (0.360) | (0.373) |
| Intervention (=1 if PEPFAR) | -2.054*** | -2.245* | -1.700* | -2.495 | -1.950*** | -2.100** |
|  | (0.515) | (0.925) | (0.761) | (1.307) | (0.444) | (0.796) |
| Interaction term (PEPFAR impact estimate) | 2.945*** | 2.805*** | 1.839 | 1.729 | 2.623*** | 2.504*** |
|  | (0.707) | (0.704) | (1.053) | (1.081) | (0.610) | (0.615) |
| Country income group (=1 if middle income) |  | n/a |  | n/a |  | n/a |
|  |  | n/a |  | n/a |  | n/a |
| BL population |  | 1.19e-09 |  | -2.75e-09 |  | 0.357 |
|  |  | (1.34e-09) |  | (6.72e-09) |  | (0.581) |
| BL per capita non-PEPFAR donor spending on health (constant $) |  | -0.00308 |  | -0.00757 |  | 1.43e-09 |
|  |  | (0.00568) |  | (0.00619) |  | (1.26e-09) |
| BL per capita domestic health spending (current $) |  | -0.00353** |  | -0.00295* |  | -0.00265 |
|  |  | (0.00123) |  | (0.00136) |  | (0.00550) |
| BL GDP per capita, PPP (constant 2011 international $) |  | 0.000176*** |  | 0.000157*** |  | -0.00333** |
|  |  | (0.0000427) |  | (0.0000457) |  | (0.00114) |
| BL HIV prevalence (% of population ages 15-49) |  | -0.0952 |  | -0.0342 |  | 0.000170*** |
|  |  | (0.0639) |  | (0.0679) |  | (0.0000413) |
| BL life expectancy at birth |  | -0.104 |  | -0.124* |  | -0.0477 |
|  |  | (0.0551) |  | (0.0616) |  | (0.0500) |
| BL urban population (%) |  | 0.00189 |  | 0.00715 |  | -0.0690 |
|  |  | (0.0108) |  | (0.0118) |  | (0.0510) |
| BL school enrollment, secondary (% gross) |  | -0.0419*** |  | -0.0300* |  | -0.00524 |
|  |  | (0.0121) |  | (0.0136) |  | (0.0102) |
| BL fertility rate (births per woman) |  | -1.200*** |  | -0.796* |  | -0.0437*** |
|  |  | (0.246) |  | (0.314) |  | (0.0115) |
| Recipient of US HIV aid before 2004 (=1 if yes) |  | 0.325 |  | 0.0690 |  | -1.061*** |
|  |  | (0.583) |  | (0.933) |  | (0.233) |
| Constant | 2.875*** | 16.15*** | 2.875*** | 15.33** | 2.875*** | 13.50*** |
|  | (0.276) | (4.476) | (0.288) | (4.923) | (0.268) | (4.097) |
| Observations | 2315 | 2051 | 1942 | 1678 | 2576 | 2283 |
| Adjusted R-squared | 0.008 | 0.026 | 0.001 | 0.011 | 0.009 | 0.025 |

Notes: Standard errors in parentheses; ***p < 0.001   **p < 0.01 * p< 0.05. Source: Our data came from four publicly available datasets: World Bank’s World Development Indicators; U.S. government’s foreignassistance.gov database; OECD Creditor Reporting System database; and the Institute of Health Metrics and Evaluation GBD Result’s Tool.

**Exhibit [E]. DID results for PEPFAR impact on GDP growth per capita for PEPFAR countries by five-year time periods**

| **Variables** | **PEPFAR countries** | | | | | | |
| --- | --- | --- | --- | --- | --- | --- | --- |
|  | **Unadjusted** | | | | **Adjusted** | | |
|  | **2004-2008** | | **2009-2013** | **2014-2018** | **2004-2008** | **2009-2013** | **2014-2018** |
| Interaction term (PEPFAR impact estimate) | 1.335* | 0.852* | | 0.0891 | 0.878 | 0.980* | 0.214 |
|  | (0.634) | (0.358) | | (0.167) | (0.631) | (0.401) | (0.180) |

**Exhibit [E]. DID results for PEPFAR impact on GDP growth per capita for COP countries by five-year time periods**

| **Variables** | **COP countries** | | | | | | |
| --- | --- | --- | --- | --- | --- | --- | --- |
|  | **Unadjusted** | | | | **Adjusted** | | |
|  | **2004-2008** | | **2009-2013** | **2014-2018** | **2004-2008** | **2009-2013** | **2014-2018** |
| Interaction term (PEPFAR impact estimate) | 1.183 | 1.387*** | | 0.0529 | 0.810 | 1.519*** | 0.174 |
|  | (0.895) | (0.384) | | (0.186) | (0.879) | (0.423) | (0.201) |

**Exhibit [E]. DID results for PEPFAR impact on GDP growth per capita by PEPFAR country income group (without India and China)**

| **Variables** | **Low-income PEPFAR countries without India** | | **Middle-income PEPFAR countries without China** | | **All PEPFAR countries without India and China** | |
| --- | --- | --- | --- | --- | --- | --- |
|  | **Unadjusted** | **Adjusted** | **Unadjusted** | **Adjusted** | **Unadjusted** | **Adjusted** |
| Time variable (=1 post-2004) | -0.287 | -0.126 | -0.287 | -0.120 | -0.287 | -0.115 |
|  | (0.354) | (0.366) | (0.358) | (0.369) | (0.333) | (0.346) |
| Intervention (=1 if PEPFAR) | -2.471*** | -2.341*** | -1.620*** | -1.223* | -2.104*** | -1.782*** |
|  | (0.386) | (0.597) | (0.422) | (0.514) | (0.319) | (0.393) |
| Interaction term (PEPFAR impact estimate) | 2.751*** | 2.675*** | 1.712** | 1.332* | 2.305*** | 2.096*** |
|  | (0.523) | (0.524) | (0.577) | (0.580) | (0.432) | (0.439) |
| Country income group (=1 if middle income) |  | n/a |  | n/a |  | 0.252 |
|  |  | n/a |  | n/a |  | (0.401) |
| BL population |  | 5.84e-09 |  | -3.52e-09 |  | 8.84e-10 |
|  |  | (4.86e-09) |  | (4.26e-09) |  | (3.08e-09) |
| BL per capita non-PEPFAR donor spending on health (constant $) |  | -0.00180 |  | -0.00692 |  | -0.00319 |
|  |  | (0.00515) |  | (0.00545) |  | (0.00478) |
| BL per capita domestic health spending (current $) |  | -0.00314** |  | -0.00299** |  | -0.00304*** |
|  |  | (0.00108) |  | (0.00105) |  | (0.000869) |
| BL GDP per capita, PPP (constant 2011 international $) |  | 0.000176*** |  | 0.000134*** |  | 0.000145*** |
|  |  | (0.0000394) |  | (0.0000360) |  | (0.0000325) |
| BL HIV prevalence (% of population ages 15-49) |  | -0.0463 |  | -0.0156 |  | -0.00260 |
|  |  | (0.0534) |  | (0.0545) |  | (0.0380) |
| BL life expectancy at birth |  | -0.0568 |  | -0.0553 |  | -0.00979 |
|  |  | (0.0415) |  | (0.0445) |  | (0.0322) |
| BL urban population (%) |  | -0.00899 |  | 0.00427 |  | -0.0125 |
|  |  | (0.00923) |  | (0.00952) |  | (0.00762) |
| BL school enrollment, secondary (% gross) |  | -0.0344*** |  | -0.0241* |  | -0.0295*** |
|  |  | (0.00984) |  | (0.0104) |  | (0.00810) |
| BL fertility rate (births per woman) |  | -0.950*** |  | -0.624** |  | -0.722*** |
|  |  | (0.196) |  | (0.214) |  | (0.153) |
| Recipient of US HIV aid before 2004 (=1 if yes) |  | 0.368 |  | -0.397 |  | 0.0728 |
|  |  | (0.360) |  | (0.487) |  | (0.278) |
| Constant | 2.875*** | 11.89*** | 2.875*** | 10.01** | 2.875*** | 7.928** |
|  | (0.263) | (3.400) | (0.266) | (3.546) | (0.248) | (2.545) |
| Observations | 3093 | 2829 | 2722 | 2424 | 4134 | 3807 |
| Adjusted R-squared | 0.017 | 0.032 | 0.005 | 0.013 | 0.016 | 0.028 |

Notes: Standard errors in parentheses; ***p < 0.001   **p < 0.01 * p< 0.05. Source: Our data came from four publicly available datasets: World Bank’s World Development Indicators; U.S. government’s foreignassistance.gov database; OECD Creditor Reporting System database; and the Institute of Health Metrics and Evaluation GBD Result’s Tool.

**Exhibit [E]. DID results for PEPFAR impact on GDP growth per capita by COP country income group (without India)**

| **Variables** | **Low-income COP countries**  **without India** | | **All COP countries**  **without India** | |
| --- | --- | --- | --- | --- |
|  | **Unadjusted** | **Adjusted** | **Unadjusted** | **Adjusted** |
| Time variable (=1 post-2004) | -0.287 | -0.129 | -0.287 | -0.125 |
|  | (0.373) | (0.386) | (0.362) | (0.376) |
| Intervention (=1 if PEPFAR) | -2.189*** | -2.333* | -2.041*** | -2.093** |
|  | (0.526) | (0.944) | (0.451) | (0.809) |
| Interaction term (PEPFAR impact estimate) | 2.977*** | 2.836*** | 2.634*** | 2.516*** |
|  | (0.723) | (0.721) | (0.619) | (0.626) |
| Country income group (=1 if middle income) |  | n/a |  | 0.364 |
|  |  | n/a |  | (0.586) |
| BL population |  | 5.51e-09 |  | 7.52e-10 |
|  |  | (8.34e-09) |  | (5.38e-09) |
| BL per capita non-PEPFAR donor spending on health (constant $) |  | -0.00260 |  | -0.00272 |
|  |  | (0.00579) |  | (0.00556) |
| BL per capita domestic health spending (current $) |  | -0.00351** |  | -0.00335** |
|  |  | (0.00124) |  | (0.00116) |
| BL GDP per capita, PPP (constant 2011 international $) |  | 0.000176*** |  | 0.000171*** |
|  |  | (0.0000430) |  | (0.0000416) |
| BL HIV prevalence (% of population ages 15-49) |  | -0.0853 |  | -0.0492 |
|  |  | (0.0670) |  | (0.0516) |
| BL life expectancy at birth |  | -0.102 |  | -0.0695 |
|  |  | (0.0556) |  | (0.0515) |
| BL urban population (%) |  | 0.000615 |  | -0.00507 |
|  |  | (0.0111) |  | (0.0103) |
| BL school enrollment, secondary (% gross) |  | -0.0424*** |  | -0.0438*** |
|  |  | (0.0122) |  | (0.0116) |
| BL fertility rate (births per woman) |  | -1.201*** |  | -1.064*** |
|  |  | (0.248) |  | (0.235) |
| Recipient of US HIV aid before 2004 (=1 if yes) |  | 0.214 |  | 0.239 |
|  |  | (0.624) |  | (0.552) |
| Constant | 2.875*** | 16.06*** | 2.875*** | 13.54** |
|  | (0.278) | (4.509) | (0.269) | (4.133) |
| Observations | 2286 | 2022 | 2547 | 2254 |
| Adjusted R-squared | 0.008 | 0.025 | 0.009 | 0.024 |

Notes: Standard errors in parentheses; ***p < 0.001   **p < 0.01 * p< 0.05. Source: Our data came from four publicly available datasets: World Bank’s World Development Indicators; U.S. government’s foreignassistance.gov database; OECD Creditor Reporting System database; and the Institute of Health Metrics and Evaluation GBD Result’s Tool.

**Exhibit [E]. DID results for PEPFAR impact on GDP growth per capita for PEPFAR countries by five-year time periods (without India and China)**

| **Variables** | **PEPFAR countries without India and China** | | | | | | |
| --- | --- | --- | --- | --- | --- | --- | --- |
|  | **Unadjusted** | | | | **Adjusted** | | |
|  | **2004-2008** | | **2009-2013** | **2014-2018** | **2004-2008** | **2009-2013** | **2014-2018** |
| Interaction term (PEPFAR impact estimate) | 1.369* | 0.848* | | 0.0880 | 0.907 | 0.976* | 0.213 |
|  | (0.639) | (0.358) | | (0.167) | (0.638) | (0.403) | (0.181) |

**Exhibit [E]. DID results for PEPFAR impact on GDP growth per capita for COP countries by five-year time periods (without India)**

| **Variables** | **COP countries without India** | | | | | | |
| --- | --- | --- | --- | --- | --- | --- | --- |
|  | **Unadjusted** | | | | **Adjusted** | | |
|  | **2004-2008** | | **2009-2013** | **2014-2018** | **2004-2008** | **2009-2013** | **2014-2018** |
| Interaction term (PEPFAR impact estimate) | 1.230 | 1.379*** | | 0.0251 | 0.863 | 1.509*** | 0.144 |
|  | (0.909) | (0.388) | | (0.188) | (0.894) | (0.427) | (0.204) |

**Exhibit [E]. DID results for PEPFAR impact on GDP growth per capita by PEPFAR country income group (with 1999-2003 as shorter pre-treatment period)**

| **Variables** | **Low-income**  **PEPFAR countries** | | **Middle-income**  **PEPFAR countries** | | **All**  **PEPFAR countries** | |
| --- | --- | --- | --- | --- | --- | --- |
|  | **Unadjusted** | **Adjusted** | **Unadjusted** | **Adjusted** | **Unadjusted** | **Adjusted** |
| Time variable (=1 post-2004) | 0.444 | 0.287 | 0.444 | 0.286 | 0.444 | 0.289 |
|  | (0.411) | (0.435) | (0.400) | (0.419) | (0.380) | (0.399) |
| Intervention (=1 if PEPFAR) | -0.710 | -0.644 | 0.997 | 0.799 | 0.0178 | -0.352 |
|  | (0.529) | (0.716) | (0.562) | (0.641) | (0.429) | (0.496) |
| Interaction term (PEPFAR impact estimate) | 1.044 | 1.277* | -0.746 | -0.729 | 0.281 | 0.439 |
|  | (0.609) | (0.623) | (0.648) | (0.659) | (0.493) | (0.506) |
| Country income group (=1 if middle income) |  | n/a |  | n/a |  | -0.0823 |
|  |  | n/a |  | n/a |  | (0.399) |
| BL population |  | 7.53e-10 |  | 2.83e-09** |  | 1.99e-09** |
|  |  | (1.25e-09) |  | (1.05e-09) |  | (7.56e-10) |
| BL per capita non-PEPFAR donor spending on health (constant $) |  | -0.00726 |  | -0.00986 |  | -0.00887 |
|  |  | (0.00534) |  | (0.00542) |  | (0.00481) |
| BL per capita domestic health spending (current $) |  | -0.00158 |  | -0.00264* |  | -0.00194* |
|  |  | (0.00110) |  | (0.00103) |  | (0.000867) |
| BL GDP per capita, PPP (constant 2011 international $) |  | 0.0000502 |  | 0.0000236 |  | 0.0000286 |
|  |  | (0.0000407) |  | (0.0000355) |  | (0.0000325) |
| BL HIV prevalence (% of population ages 15-49) |  | -0.163** |  | -0.144** |  | -0.131*** |
|  |  | (0.0556) |  | (0.0541) |  | (0.0381) |
| BL life expectancy at birth |  | -0.116** |  | -0.178*** |  | -0.111*** |
|  |  | (0.0427) |  | (0.0441) |  | (0.0323) |
| BL urban population (%) |  | -0.0116 |  | 0.00566 |  | -0.0131 |
|  |  | (0.00952) |  | (0.00936) |  | (0.00763) |
| BL school enrollment, secondary (% gross) |  | -0.0119 |  | -0.0117 |  | -0.00856 |
|  |  | (0.0101) |  | (0.0103) |  | (0.00814) |
| BL fertility rate (births per woman) |  | -1.184*** |  | -1.497*** |  | -1.143*** |
|  |  | (0.200) |  | (0.212) |  | (0.152) |
| Recipient of US HIV aid before 2004 (=1 if yes) |  | 0.684 |  | -0.743 |  | 0.242 |
|  |  | (0.371) |  | (0.462) |  | (0.274) |
| Constant | 2.144*** | 15.48*** | 2.144*** | 20.65*** | 2.144*** | 15.35*** |
|  | (0.358) | (3.497) | (0.348) | (3.525) | (0.331) | (2.544) |
| Observations | 2248 | 2038 | 1971 | 1736 | 2993 | 2738 |
| Adjusted R-squared | 0.004 | 0.029 | 0.001 | 0.041 | 0.002 | 0.038 |

Notes: Standard errors in parentheses; ***p < 0.001   **p < 0.01 * p< 0.05. Source: Our data came from four publicly available datasets: World Bank’s World Development Indicators; U.S. government’s foreignassistance.gov database; OECD Creditor Reporting System database; and the Institute of Health Metrics and Evaluation GBD Result’s Tool.

**Exhibit [E]. DID results for PEPFAR impact on GDP growth per capita by COP country income group (with 1999-2003 as shorter pre-treatment period)**

| **Variables** | **Low-income**  **COP countries** | | **Middle-income**  **COP countries** | | **All**  **COP countries** | |
| --- | --- | --- | --- | --- | --- | --- |
|  | **Unadjusted** | **Adjusted** | **Unadjusted** | **Adjusted** | **Unadjusted** | **Adjusted** |
| Time variable (=1 post-2004) | 0.444 | 0.285 | 0.444 | 0.282 | 0.444 | 0.286 |
|  | (0.421) | (0.444) | (0.442) | (0.474) | (0.407) | (0.431) |
| Intervention (=1 if PEPFAR) | -0.524 | -0.485 | 0.288 | -0.688 | -0.288 | -0.726 |
|  | (0.704) | (1.063) | (1.061) | (1.565) | (0.605) | (0.916) |
| Interaction term (PEPFAR impact estimate) | 1.414 | 1.597 | -0.149 | -0.0397 | 0.961 | 1.144 |
|  | (0.812) | (0.826) | (1.224) | (1.293) | (0.697) | (0.718) |
| Country income group (=1 if middle income) |  | n/a |  | n/a |  | -0.152 |
|  |  | n/a |  | n/a |  | (0.588) |
| BL population |  | -2.92e-10 |  | -5.96e-09 |  | 2.02e-10 |
|  |  | (1.37e-09) |  | (6.98e-09) |  | (1.28e-09) |
| BL per capita non-PEPFAR donor spending on health (constant $) |  | -0.00717 |  | -0.00993 |  | -0.00725 |
|  |  | (0.00578) |  | (0.00640) |  | (0.00558) |
| BL per capita domestic health spending (current $) |  | -0.00232 |  | -0.00241 |  | -0.00185 |
|  |  | (0.00122) |  | (0.00138) |  | (0.00114) |
| BL GDP per capita, PPP (constant 2011 international $) |  | 0.0000582 |  | 0.0000522 |  | 0.0000480 |
|  |  | (0.0000430) |  | (0.0000468) |  | (0.0000414) |
| BL HIV prevalence (% of population ages 15-49) |  | -0.247*** |  | -0.154* |  | -0.178*** |
|  |  | (0.0651) |  | (0.0702) |  | (0.0507) |
| BL life expectancy at birth |  | -0.186*** |  | -0.224*** |  | -0.162** |
|  |  | (0.0561) |  | (0.0634) |  | (0.0517) |
| BL urban population (%) |  | 0.00303 |  | 0.0106 |  | -0.000842 |
|  |  | (0.0109) |  | (0.0121) |  | (0.0102) |
| BL school enrollment, secondary (% gross) |  | -0.0267* |  | -0.0199 |  | -0.0281* |
|  |  | (0.0123) |  | (0.0141) |  | (0.0116) |
| BL fertility rate (births per woman) |  | -1.657*** |  | -1.626*** |  | -1.511*** |
|  |  | (0.249) |  | (0.323) |  | (0.235) |
| Recipient of US HIV aid before 2004 (=1 if yes) |  | 0.634 |  | 0.199 |  | 0.418 |
|  |  | (0.596) |  | (0.970) |  | (0.545) |
| Constant | 2.144*** | 22.33*** | 2.144*** | 24.21*** | 2.144*** | 20.62*** |
|  | (0.366) | (4.555) | (0.385) | (5.074) | (0.354) | (4.146) |
| Observations | 1666 | 1456 | 1406 | 1196 | 1846 | 1616 |
| Adjusted R-squared | 0.005 | 0.037 | -0.001 | 0.018 | 0.003 | 0.032 |

Notes: Standard errors in parentheses; ***p < 0.001   **p < 0.01 * p< 0.05. Source: Our data came from four publicly available datasets: World Bank’s World Development Indicators; U.S. government’s foreignassistance.gov database; OECD Creditor Reporting System database; and the Institute of Health Metrics and Evaluation GBD Result’s Tool.

**Exhibit [E]. DID results for PEPFAR impact on GDP growth per capita for PEPFAR countries by five-year time periods (with 1999-2003 as shorter pre-treatment period)**

| **Variables** | **PEPFAR countries without India and China** | | | | | | |
| --- | --- | --- | --- | --- | --- | --- | --- |
|  | **Unadjusted** | | | | **Adjusted** | | |
|  | **2004-2008** | | **2009-2013** | **2014-2018** | **2004-2008** | **2009-2013** | **2014-2018** |
| Interaction term (PEPFAR impact estimate) | -0.660 | 0.852* | | 0.0891 | -0.746 | 0.978* | 0.207 |
|  | (0.584) | (0.358) | | (0.167) | (0.589) | (0.396) | (0.175) |

**Exhibit []. DID results for PEPFAR impact on GDP growth per capita for COP countries by five-year time periods (with 1999-2003 as shorter pre-treatment period)**

| **Variables** | **COP countries without India** | | | | | | |
| --- | --- | --- | --- | --- | --- | --- | --- |
|  | **Unadjusted** | | | | **Adjusted** | | |
|  | **2004-2008** | | **2009-2013** | **2014-2018** | **2004-2008** | **2009-2013** | **2014-2018** |
| Interaction term (PEPFAR impact estimate) | -0.480 | 1.387*** | | 0.0529 | -0.550 | 1.529*** | 0.165 |
|  | (0.780) | (0.384) | | (0.186) | (0.767) | (0.425) | (0.200) |

**Exhibit F. Sensitivity test for PEPFAR impact on female primary school disengagement**

- by PEPFAR/COP country income group
- by three five-year intervals of the post-treatment period for PEPFAR/COP country
- exclude China and India as PEPFAR/COP country

**Exhibit [F]. DID results for PEPFAR impact on female primary school disengagement rate by PEPFAR country income group**

| **Variables** | **Low-income**  **PEPFAR countries** | | **Middle-income**  **PEPFAR countries** | | **All**  **PEPFAR countries** | |
| --- | --- | --- | --- | --- | --- | --- |
|  | **Unadjusted** | **Adjusted** | **Unadjusted** | **Adjusted** | **Unadjusted** | **Adjusted** |
| Time variable (=1 post-2004) | -4.915*** | -4.260*** | -4.915*** | -4.656*** | -4.915*** | -4.551*** |
|  | (1.207) | (0.947) | (0.969) | (0.598) | (1.365) | (0.928) |
| Intervention (=1 if PEPFAR) | 31.14*** | 9.456*** | 1.821 | -4.622*** | 19.37*** | 5.895*** |
|  | (1.302) | (1.472) | (1.164) | (0.842) | (1.320) | (1.049) |
| Interaction term (PEPFAR impact estimate) | -16.30*** | -16.86*** | 1.506 | 3.436*** | -9.271*** | -9.185*** |
|  | (1.693) | (1.286) | (1.506) | (0.920) | (1.713) | (1.143) |
| Country income group (=1 if middle income) |  | n/a |  | n/a |  | 0.334 |
|  |  | n/a |  | n/a |  | (1.030) |
| BL population |  | -4.23e-09 |  | -1.74e-09 |  | -4.55e-09* |
|  |  | (2.84e-09) |  | (1.99e-09) |  | (2.08e-09) |
| BL per capita non-PEPFAR donor spending on health (constant $) |  | -0.0844*** |  | -0.00725 |  | -0.0884*** |
|  |  | (0.0234) |  | (0.0159) |  | (0.0225) |
| BL per capita domestic health spending (current $) |  | 0.00272 |  | 0.00338* |  | 0.00771*** |
|  |  | (0.00260) |  | (0.00161) |  | (0.00218) |
| BL GDP per capita, PPP (constant 2011 international $) |  | 0.000272** |  | 0.000206*** |  | 0.0000150 |
|  |  | (0.0000986) |  | (0.0000571) |  | (0.0000843) |
| BL HIV prevalence (% of population ages 15-49) |  | -1.471*** |  | -0.334*** |  | -1.008*** |
|  |  | (0.120) |  | (0.0806) |  | (0.0920) |
| BL life expectancy at birth |  | -0.938*** |  | -0.427*** |  | -0.770*** |
|  |  | (0.108) |  | (0.0723) |  | (0.0891) |
| BL urban population (%) |  | -0.00676 |  | -0.0328* |  | -0.00774 |
|  |  | (0.0254) |  | (0.0163) |  | (0.0214) |
| BL school enrollment, secondary (% gross) |  | -0.221*** |  | -0.158*** |  | -0.170*** |
|  |  | (0.0269) |  | (0.0186) |  | (0.0221) |
| BL fertility rate (births per woman) |  | 2.765*** |  | 2.490*** |  | 3.558*** |
|  |  | (0.492) |  | (0.356) |  | (0.388) |
| Recipient of US HIV aid before 2004 (=1 if yes) |  | -1.582* |  | 2.832*** |  | -1.015 |
|  |  | (0.796) |  | (0.683) |  | (0.662) |
| Constant | 11.54*** | 85.82*** | 11.54*** | 46.16*** | 11.54*** | 68.15*** |
|  | (0.931) | (8.496) | (0.747) | (5.475) | (1.053) | (6.647) |
| Observations | 1237 | 1169 | 1041 | 950 | 1669 | 1577 |
| Adjusted R-squared | 0.448 | 0.715 | 0.041 | 0.440 | 0.220 | 0.669 |

Notes: Standard errors in parentheses; ***p < 0.001   **p < 0.01 * p< 0.05. Source: Our data came from four publicly available datasets: World Bank’s World Development Indicators; U.S. government’s foreignassistance.gov database; OECD Creditor Reporting System database; and the Institute of Health Metrics and Evaluation GBD Result’s Tool.

**Exhibit [F]. DID results for PEPFAR impact on female primary school disengagement rate by COP country income group**

| **Variables** | **Low-income**  **COP countries** | | **Middle-income**  **COP countries** | | **All**  **COP countries** | | |
| --- | --- | --- | --- | --- | --- | --- | --- |
|  | **Unadjusted** | **Adjusted** | **Unadjusted** | **Adjusted** | | **Unadjusted** | **Adjusted** |
| Time variable (=1 post-2004) | -4.915*** | -4.163*** | -4.915*** | -4.143*** | | -4.915*** | -4.113*** |
|  | (0.955) | (0.812) | (0.791) | (0.619) | | (1.019) | (0.835) |
| Intervention (=1 if PEPFAR) | 27.32*** | 2.706 | -0.0521 | -9.308*** | | 18.31*** | 1.598 |
|  | (1.335) | (2.155) | (1.451) | (1.821) | | (1.250) | (1.735) |
| Interaction term (PEPFAR impact estimate) | -18.90*** | -19.86*** | 3.305 | 2.572 | | -11.60*** | -12.58*** |
|  | (1.777) | (1.433) | (1.936) | (1.450) | | (1.658) | (1.304) |
| Country income group (=1 if middle income) |  | n/a |  | n/a | |  | 0.0606 |
|  |  | n/a |  | n/a | |  | (1.307) |
| BL population |  | -1.43e-09 |  | -3.20e-08*** | |  | -4.33e-09 |
|  |  | (2.69e-09) |  | (7.98e-09) | |  | (2.62e-09) |
| BL per capita non-PEPFAR donor spending on health (constant $) |  | -0.0538* |  | -0.0200 | |  | -0.0488* |
|  |  | (0.0216) |  | (0.0170) | |  | (0.0221) |
| BL per capita domestic health spending (current $) |  | 0.00269 |  | -0.000715 | |  | 0.000716 |
|  |  | (0.00250) |  | (0.00200) | |  | (0.00236) |
| BL GDP per capita, PPP (constant 2011 international $) |  | 0.000286** |  | 0.000404*** | |  | 0.000317*** |
|  |  | (0.0000900) |  | (0.0000708) | |  | (0.0000896) |
| BL HIV prevalence (% of population ages 15-49) |  | -0.910*** |  | -0.523*** | |  | -0.747*** |
|  |  | (0.120) |  | (0.0964) | |  | (0.106) |
| BL life expectancy at birth |  | -0.878*** |  | -0.749*** | |  | -0.795*** |
|  |  | (0.119) |  | (0.0922) | |  | (0.120) |
| BL urban population (%) |  | -0.0286 |  | -0.0234 | |  | -0.0107 |
|  |  | (0.0251) |  | (0.0197) | |  | (0.0245) |
| BL school enrollment, secondary (% gross) |  | -0.160*** |  | -0.183*** | |  | -0.125*** |
|  |  | (0.0300) |  | (0.0243) | |  | (0.0278) |
| BL fertility rate (births per woman) |  | 2.780*** |  | 1.369* | |  | 2.725*** |
|  |  | (0.542) |  | (0.530) | |  | (0.520) |
| Recipient of US HIV aid before 2004 (=1 if yes) |  | 4.179*** |  | 8.131*** | |  | 4.228*** |
|  |  | (1.149) |  | (1.108) | |  | (1.093) |
| Constant | 11.54*** | 77.06*** | 11.54*** | 72.90*** | | 11.54*** | 67.94*** |
|  | (0.736) | (9.365) | (0.610) | (7.087) | | (0.785) | (9.077) |
| Observations | 850 | 782 | 728 | 661 | | 969 | 901 |
| Adjusted R-squared | 0.435 | 0.668 | 0.053 | 0.537 | | 0.291 | 0.609 |

Notes: Standard errors in parentheses; ***p < 0.001   **p < 0.01 * p< 0.05. Source: Our data came from four publicly available datasets: World Bank’s World Development Indicators; U.S. government’s foreignassistance.gov database; OECD Creditor Reporting System database; and the Institute of Health Metrics and Evaluation GBD Result’s Tool.

**Exhibit [F]. DID results for PEPFAR impact on female primary school disengagement rate for PEPFAR countries by five-year time periods**

| **Variables** | **PEPFAR countries** | | | | | | |
| --- | --- | --- | --- | --- | --- | --- | --- |
|  | **Unadjusted** | | | | **Adjusted** | | |
|  | **2004-2008** | | **2009-2013** | **2014-2018** | **2004-2008** | **2009-2013** | **2014-2018** |
| Interaction term (PEPFAR impact estimate) | -7.421** | -1.327 | | -0.523 | -6.695*** | -1.514* | -0.976* |
|  | (2.568) | (0.914) | | (0.418) | (1.475) | (0.722) | (0.388) |

**Exhibit [F]. DID results for PEPFAR impact on female primary school disengagement rate for COP countries by five-year time periods**

| **Variables** | **COP countries** | | | | | | |
| --- | --- | --- | --- | --- | --- | --- | --- |
|  | **Unadjusted** | | | | **Adjusted** | | |
|  | **2004-2008** | | **2009-2013** | **2014-2018** | **2004-2008** | **2009-2013** | **2014-2018** |
| Interaction term (PEPFAR impact estimate) | -9.142*** | -1.765 | | -0.695 | -9.669*** | -1.690 | -1.223* |
|  | (2.476) | (1.037) | | (0.425) | (1.770) | (1.127) | (0.538) |

**Exhibit [F]. DID results for PEPFAR impact on female primary school disengagement rate by PEPFAR country income group (without India and China)**

| **Variables** | **Low-income PEPFAR countries without India** | | **Middle-income PEPFAR countries without China** | | **All PEPFAR countries without India and China** | |
| --- | --- | --- | --- | --- | --- | --- |
|  | **Unadjusted** | **Adjusted** | **Unadjusted** | **Adjusted** | **Unadjusted** | **Adjusted** |
| Time variable (=1 post-2004) | -4.915*** | -4.301*** | -4.915*** | -4.551*** | -4.915*** | -4.584*** |
|  | (1.202) | (0.940) | (0.972) | (0.593) | (1.365) | (0.933) |
| Intervention (=1 if PEPFAR) | 31.53*** | 9.732*** | 2.003 | -4.752*** | 19.83*** | 5.742*** |
|  | (1.303) | (1.465) | (1.182) | (0.835) | (1.327) | (1.058) |
| Interaction term (PEPFAR impact estimate) | -16.32*** | -17.10*** | 1.323 | 3.507*** | -9.573*** | -9.058*** |
|  | (1.694) | (1.283) | (1.522) | (0.911) | (1.719) | (1.152) |
| Country income group (=1 if middle income) |  | n/a |  | n/a |  | 0.00808 |
|  |  | n/a |  | n/a |  | (1.063) |
| BL population |  | 5.59e-08*** |  | -2.95e-08*** |  | 2.90e-09 |
|  |  | (1.21e-08) |  | (5.96e-09) |  | (7.70e-09) |
| BL per capita non-PEPFAR donor spending on health (constant $) |  | -0.0667** |  | -0.0154 |  | -0.0852*** |
|  |  | (0.0235) |  | (0.0158) |  | (0.0228) |
| BL per capita domestic health spending (current $) |  | 0.00289 |  | 0.00226 |  | 0.00809*** |
|  |  | (0.00258) |  | (0.00161) |  | (0.00221) |
| BL GDP per capita, PPP (constant 2011 international $) |  | 0.000264** |  | 0.000242*** |  | 0.00000806 |
|  |  | (0.0000979) |  | (0.0000571) |  | (0.0000852) |
| BL HIV prevalence (% of population ages 15-49) |  | -1.429*** |  | -0.416*** |  | -0.988*** |
|  |  | (0.119) |  | (0.0815) |  | (0.0945) |
| BL life expectancy at birth |  | -0.941*** |  | -0.496*** |  | -0.754*** |
|  |  | (0.108) |  | (0.0730) |  | (0.0910) |
| BL urban population (%) |  | -0.0131 |  | -0.0261 |  | -0.00915 |
|  |  | (0.0253) |  | (0.0162) |  | (0.0215) |
| BL school enrollment, secondary (% gross) |  | -0.205*** |  | -0.155*** |  | -0.167*** |
|  |  | (0.0269) |  | (0.0184) |  | (0.0223) |
| BL fertility rate (births per woman) |  | 2.850*** |  | 2.293*** |  | 3.623*** |
|  |  | (0.489) |  | (0.355) |  | (0.394) |
| Recipient of US HIV aid before 2004 (=1 if yes) |  | -1.988* |  | 3.667*** |  | -1.082 |
|  |  | (0.794) |  | (0.697) |  | (0.673) |
| Constant | 11.54*** | 83.87*** | 11.54*** | 51.54*** | 11.54*** | 66.80*** |
|  | (0.927) | (8.445) | (0.749) | (5.531) | (1.052) | (6.814) |
| Observations | 1225 | 1157 | 1034 | 943 | 1650 | 1558 |
| Adjusted R-squared | 0.456 | 0.720 | 0.042 | 0.455 | 0.226 | 0.668 |

Notes: Standard errors in parentheses; ***p < 0.001   **p < 0.01 * p< 0.05. Source: Our data came from four publicly available datasets: World Bank’s World Development Indicators; U.S. government’s foreignassistance.gov database; OECD Creditor Reporting System database; and the Institute of Health Metrics and Evaluation GBD Result’s Tool.

**Exhibit [F]. DID results for PEPFAR impact on female primary school disengagement rate by COP country income group (without India)**

| **Variables** | **Low-income COP countries**  **without India** | | **All COP countries**  **without India** | |
| --- | --- | --- | --- | --- |
|  | **Unadjusted** | **Adjusted** | **Unadjusted** | **Adjusted** |
| Time variable (=1 post-2004) | -4.915*** | -4.289*** | -4.915*** | -4.109*** |
|  | (0.950) | (0.806) | (1.020) | (0.840) |
| Intervention (=1 if PEPFAR) | 28.10*** | 2.252 | 18.54*** | 1.554 |
|  | (1.350) | (2.149) | (1.264) | (1.792) |
| Interaction term (PEPFAR impact estimate) | -18.98*** | -20.06*** | -11.43*** | -12.46*** |
|  | (1.800) | (1.448) | (1.678) | (1.326) |
| Country income group (=1 if middle income) |  | n/a |  | 0.0978 |
|  |  | n/a |  | (1.367) |
| BL population |  | 7.26e-08*** |  | -5.35e-09 |
|  |  | (1.67e-08) |  | (9.79e-09) |
| BL per capita non-PEPFAR donor spending on health (constant $) |  | -0.0348 |  | -0.0491* |
|  |  | (0.0218) |  | (0.0223) |
| BL per capita domestic health spending (current $) |  | 0.00296 |  | 0.000678 |
|  |  | (0.00248) |  | (0.00240) |
| BL GDP per capita, PPP (constant 2011 international $) |  | 0.000288** |  | 0.000317*** |
|  |  | (0.0000893) |  | (0.0000905) |
| BL HIV prevalence (% of population ages 15-49) |  | -0.768*** |  | -0.750*** |
|  |  | (0.124) |  | (0.112) |
| BL life expectancy at birth |  | -0.807*** |  | -0.797*** |
|  |  | (0.119) |  | (0.122) |
| BL urban population (%) |  | -0.0454 |  | -0.0107 |
|  |  | (0.0251) |  | (0.0247) |
| BL school enrollment, secondary (% gross) |  | -0.179*** |  | -0.125*** |
|  |  | (0.0300) |  | (0.0280) |
| BL fertility rate (births per woman) |  | 2.598*** |  | 2.718*** |
|  |  | (0.540) |  | (0.529) |
| Recipient of US HIV aid before 2004 (=1 if yes) |  | 2.838* |  | 4.251*** |
|  |  | (1.178) |  | (1.107) |
| Constant | 11.54*** | 73.48*** | 11.54*** | 68.11*** |
|  | (0.732) | (9.328) | (0.787) | (9.260) |
| Observations | 838 | 770 | 957 | 889 |
| Adjusted R-squared | 0.444 | 0.675 | 0.292 | 0.607 |

Notes: Standard errors in parentheses; ***p < 0.001   **p < 0.01 * p< 0.05. Source: Our data came from four publicly available datasets: World Bank’s World Development Indicators; U.S. government’s foreignassistance.gov database; OECD Creditor Reporting System database; and the Institute of Health Metrics and Evaluation GBD Result’s Tool.

**Exhibit [F]. DID results for PEPFAR impact on female primary school disengagement rate for PEPFAR countries by five-year time periods (without India and China)**

| **Variables** | **PEPFAR countries without India and China** | | | | | | |
| --- | --- | --- | --- | --- | --- | --- | --- |
|  | **Unadjusted** | | | | **Adjusted** | | |
|  | **2004-2008** | | **2009-2013** | **2014-2018** | **2004-2008** | **2009-2013** | **2014-2018** |
| Interaction term (PEPFAR impact estimate) | -7.740** | -1.228 | | -0.605 | -6.501*** | -1.498* | -1.058** |
|  | (2.577) | (0.913) | | (0.423) | (1.483) | (0.728) | (0.397) |

**Exhibit [F]. DID results for PEPFAR impact on female primary school disengagement rate for COP countries by five-year time periods (without India)**

| **Variables** | **COP countries without India** | | | | | | |
| --- | --- | --- | --- | --- | --- | --- | --- |
|  | **Unadjusted** | | | | **Adjusted** | | |
|  | **2004-2008** | | **2009-2013** | **2014-2018** | **2004-2008** | **2009-2013** | **2014-2018** |
| Interaction term (PEPFAR impact estimate) | -9.002*** | -1.538 | | -0.891* | -9.522*** | -1.708 | -1.233* |
|  | (2.502) | (1.030) | | (0.444) | (1.789) | (1.151) | (0.553) |

**Exhibit G. Sensitivity test for PEPFAR impact on male primary school disengagement**

- by PEPFAR/COP country income group
- by three five-year intervals of the post-treatment period for PEPFAR/COP country
- exclude China and India as PEPFAR/COP country

**Exhibit [G]. DID results for PEPFAR impact on male primary school disengagement rate by PEPFAR country income group**

| **Variables** | **Low-income**  **PEPFAR countries** | | **Middle-income**  **PEPFAR countries** | | **All**  **PEPFAR countries** | |
| --- | --- | --- | --- | --- | --- | --- |
|  | **Unadjusted** | **Adjusted** | **Unadjusted** | **Adjusted** | **Unadjusted** | **Adjusted** |
| Time variable (=1 post-2004) | -3.010** | -2.564** | -3.010*** | -2.886*** | -3.010** | -2.863*** |
|  | (1.008) | (0.870) | (0.859) | (0.540) | (1.132) | (0.837) |
| Intervention (=1 if PEPFAR) | 25.83*** | 11.29*** | 4.091*** | -0.817 | 17.10*** | 7.023*** |
|  | (1.087) | (1.352) | (1.032) | (0.760) | (1.094) | (0.945) |
| Interaction term (PEPFAR impact estimate) | -13.80*** | -14.02*** | -0.0901 | 1.653* | -8.382*** | -7.962*** |
|  | (1.413) | (1.181) | (1.335) | (0.830) | (1.420) | (1.031) |
| Country income group (=1 if middle income) |  | n/a |  | n/a |  | -1.669 |
|  |  | n/a |  | n/a |  | (0.928) |
| BL population |  | -6.63e-09* |  | -5.24e-09** |  | -6.56e-09*** |
|  |  | (2.61e-09) |  | (1.79e-09) |  | (1.88e-09) |
| BL per capita non-PEPFAR donor spending on health (constant $) |  | -0.0140 |  | 0.0327* |  | -0.0151 |
|  |  | (0.0215) |  | (0.0143) |  | (0.0203) |
| BL per capita domestic health spending (current $) |  | -0.000348 |  | 0.00000865 |  | 0.00401* |
|  |  | (0.00239) |  | (0.00145) |  | (0.00196) |
| BL GDP per capita, PPP (constant 2011 international $) |  | 0.000414*** |  | 0.000282*** |  | 0.000163* |
|  |  | (0.0000905) |  | (0.0000516) |  | (0.0000760) |
| BL HIV prevalence (% of population ages 15-49) |  | -0.671*** |  | -0.0385 |  | -0.343*** |
|  |  | (0.110) |  | (0.0727) |  | (0.0830) |
| BL life expectancy at birth |  | -0.462*** |  | -0.271*** |  | -0.319*** |
|  |  | (0.0996) |  | (0.0652) |  | (0.0804) |
| BL urban population (%) |  | -0.00421 |  | 0.00162 |  | 0.0137 |
|  |  | (0.0234) |  | (0.0147) |  | (0.0193) |
| BL school enrollment, secondary (% gross) |  | -0.205*** |  | -0.126*** |  | -0.158*** |
|  |  | (0.0247) |  | (0.0168) |  | (0.0199) |
| BL fertility rate (births per woman) |  | 2.092*** |  | 1.589*** |  | 2.853*** |
|  |  | (0.452) |  | (0.321) |  | (0.350) |
| Recipient of US HIV aid before 2004 (=1 if yes) |  | -1.856* |  | 1.120 |  | -1.145 |
|  |  | (0.731) |  | (0.616) |  | (0.596) |
| Constant | 9.054*** | 48.94*** | 9.054*** | 30.91*** | 9.054*** | 34.46*** |
|  | (0.777) | (7.805) | (0.662) | (4.939) | (0.873) | (5.992) |
| Observations | 1237 | 1169 | 1041 | 950 | 1669 | 1577 |
| Adjusted R-squared | 0.433 | 0.645 | 0.051 | 0.374 | 0.225 | 0.598 |

Notes: Standard errors in parentheses; ***p < 0.001   **p < 0.01 * p< 0.05. Source: Our data came from four publicly available datasets: World Bank’s World Development Indicators; U.S. government’s foreignassistance.gov database; OECD Creditor Reporting System database; and the Institute of Health Metrics and Evaluation GBD Result’s Tool.

**Exhibit [G]. DID results for PEPFAR impact on male primary school disengagement rate by COP country income group**

| **Variables** | **Low-income**  **COP countries** | | **Middle-income**  **COP countries** | | **All**  **COP countries** | | |
| --- | --- | --- | --- | --- | --- | --- | --- |
|  | **Unadjusted** | **Adjusted** | **Unadjusted** | **Adjusted** | | **Unadjusted** | **Adjusted** |
| Time variable (=1 post-2004) | -3.010*** | -2.528*** | -3.010*** | -2.474*** | | -3.010*** | -2.577*** |
|  | (0.805) | (0.735) | (0.664) | (0.536) | | (0.855) | (0.749) |
| Intervention (=1 if PEPFAR) | 25.30*** | 7.986*** | 3.504** | -2.565 | | 18.12*** | 5.782*** |
|  | (1.125) | (1.950) | (1.218) | (1.577) | | (1.049) | (1.557) |
| Interaction term (PEPFAR impact estimate) | -17.96*** | -18.39*** | -0.857 | -0.970 | | -12.34*** | -12.51*** |
|  | (1.498) | (1.297) | (1.626) | (1.256) | | (1.392) | (1.171) |
| Country income group (=1 if middle income) |  | n/a |  | n/a | |  | -2.697* |
|  |  | n/a |  | n/a | |  | (1.173) |
| BL population |  | -4.44e-09 |  | -4.62e-08*** | |  | -7.41e-09** |
|  |  | (2.44e-09) |  | (6.92e-09) | |  | (2.36e-09) |
| BL per capita non-PEPFAR donor spending on health (constant $) |  | -0.00732 |  | 0.0266 | |  | 0.00248 |
|  |  | (0.0195) |  | (0.0147) | |  | (0.0198) |
| BL per capita domestic health spending (current $) |  | -0.000953 |  | -0.00665*** | |  | -0.00226 |
|  |  | (0.00226) |  | (0.00174) | |  | (0.00212) |
| BL GDP per capita, PPP (constant 2011 international $) |  | 0.000429*** |  | 0.000599*** | |  | 0.000460*** |
|  |  | (0.0000814) |  | (0.0000614) | |  | (0.0000804) |
| BL HIV prevalence (% of population ages 15-49) |  | -0.460*** |  | -0.271** | |  | -0.317*** |
|  |  | (0.109) |  | (0.0835) | |  | (0.0947) |
| BL life expectancy at birth |  | -0.648*** |  | -0.626*** | |  | -0.510*** |
|  |  | (0.108) |  | (0.0799) | |  | (0.108) |
| BL urban population (%) |  | -0.0369 |  | 0.00644 | |  | -0.0134 |
|  |  | (0.0227) |  | (0.0171) | |  | (0.0220) |
| BL school enrollment, secondary (% gross) |  | -0.130*** |  | -0.167*** | |  | -0.100*** |
|  |  | (0.0271) |  | (0.0210) | |  | (0.0249) |
| BL fertility rate (births per woman) |  | 1.586** |  | -0.741 | |  | 1.584*** |
|  |  | (0.491) |  | (0.459) | |  | (0.467) |
| Recipient of US HIV aid before 2004 (=1 if yes) |  | 1.289 |  | 4.645*** | |  | 1.723 |
|  |  | (1.040) |  | (0.959) | |  | (0.981) |
| Constant | 9.054*** | 59.19*** | 9.054*** | 64.72*** | | 9.054*** | 48.40*** |
|  | (0.620) | (8.474) | (0.512) | (6.139) | | (0.659) | (8.146) |
| Observations | 850 | 782 | 728 | 661 | | 969 | 901 |
| Adjusted R-squared | 0.460 | 0.633 | 0.052 | 0.502 | | 0.331 | 0.577 |

Notes: Standard errors in parentheses; ***p < 0.001   **p < 0.01 * p< 0.05. Source: Our data came from four publicly available datasets: World Bank’s World Development Indicators; U.S. government’s foreignassistance.gov database; OECD Creditor Reporting System database; and the Institute of Health Metrics and Evaluation GBD Result’s Tool.

**Exhibit [G]. DID results for PEPFAR impact on male primary school disengagement rate for PEPFAR countries by five-year time periods**

| **Variables** | **PEPFAR countries** | | | | | | |
| --- | --- | --- | --- | --- | --- | --- | --- |
|  | **Unadjusted** | | | | **Adjusted** | | |
|  | **2004-2008** | | **2009-2013** | **2014-2018** | **2004-2008** | **2009-2013** | **2014-2018** |
| Interaction term (PEPFAR impact estimate) | -6.769** | -1.275 | | -0.338 | -5.663*** | -1.514* | -0.786* |
|  | (2.109) | (0.782) | | (0.373) | (1.382) | (0.674) | (0.340) |

**Exhibit [G]. DID results for PEPFAR impact on male primary school disengagement rate for COP countries by five-year time periods**

| **Variables** | **COP countries** | | | | | | |
| --- | --- | --- | --- | --- | --- | --- | --- |
|  | **Unadjusted** | | | | **Adjusted** | | |
|  | **2004-2008** | | **2009-2013** | **2014-2018** | **2004-2008** | **2009-2013** | **2014-2018** |
| Interaction term (PEPFAR impact estimate) | -9.525*** | -1.995* | | -0.823* | -9.053*** | -2.050* | -1.406** |
|  | (2.037) | (0.852) | | (0.376) | (1.615) | (0.965) | (0.462) |

**Exhibit [G]. DID results for PEPFAR impact on male primary school disengagement rate by PEPFAR country income group (without India and China)**

| **Variables** | **Low-income PEPFAR countries without India** | | **Middle-income PEPFAR countries without China** | | **All PEPFAR countries without India and China** | |
| --- | --- | --- | --- | --- | --- | --- |
|  | **Unadjusted** | **Adjusted** | **Unadjusted** | **Adjusted** | **Unadjusted** | **Adjusted** |
| Time variable (=1 post-2004) | -3.010** | -2.580** | -3.010*** | -2.764*** | -3.010** | -2.855*** |
|  | (1.001) | (0.872) | (0.859) | (0.530) | (1.129) | (0.842) |
| Intervention (=1 if PEPFAR) | 26.30*** | 11.52*** | 4.433*** | -0.969 | 17.63*** | 7.026*** |
|  | (1.085) | (1.357) | (1.045) | (0.746) | (1.097) | (0.955) |
| Interaction term (PEPFAR impact estimate) | -14.01*** | -14.31*** | -0.433 | 1.735* | -8.799*** | -7.983*** |
|  | (1.410) | (1.189) | (1.346) | (0.814) | (1.422) | (1.040) |
| Country income group (=1 if middle income) |  | n/a |  | n/a |  | -1.676 |
|  |  | n/a |  | n/a |  | (0.960) |
| BL population |  | 1.69e-08 |  | -3.75e-08*** |  | -8.95e-09 |
|  |  | (1.12e-08) |  | (5.33e-09) |  | (6.95e-09) |
| BL per capita non-PEPFAR donor spending on health (constant $) |  | -0.00710 |  | 0.0232 |  | -0.0161 |
|  |  | (0.0218) |  | (0.0141) |  | (0.0206) |
| BL per capita domestic health spending (current $) |  | -0.000295 |  | -0.00129 |  | 0.00393* |
|  |  | (0.00240) |  | (0.00144) |  | (0.00199) |
| BL GDP per capita, PPP (constant 2011 international $) |  | 0.000411*** |  | 0.000324*** |  | 0.000167* |
|  |  | (0.0000907) |  | (0.0000510) |  | (0.0000768) |
| BL HIV prevalence (% of population ages 15-49) |  | -0.656*** |  | -0.134 |  | -0.350*** |
|  |  | (0.111) |  | (0.0729) |  | (0.0853) |
| BL life expectancy at birth |  | -0.463*** |  | -0.351*** |  | -0.325*** |
|  |  | (0.0998) |  | (0.0652) |  | (0.0821) |
| BL urban population (%) |  | -0.00663 |  | 0.00940 |  | 0.0140 |
|  |  | (0.0234) |  | (0.0145) |  | (0.0194) |
| BL school enrollment, secondary (% gross) |  | -0.199*** |  | -0.123*** |  | -0.158*** |
|  |  | (0.0249) |  | (0.0164) |  | (0.0201) |
| BL fertility rate (births per woman) |  | 2.125*** |  | 1.360*** |  | 2.836*** |
|  |  | (0.453) |  | (0.317) |  | (0.356) |
| Recipient of US HIV aid before 2004 (=1 if yes) |  | -2.028** |  | 2.089*** |  | -1.098 |
|  |  | (0.736) |  | (0.623) |  | (0.607) |
| Constant | 9.054*** | 48.18*** | 9.054*** | 37.16*** | 9.054*** | 34.92*** |
|  | (0.772) | (7.827) | (0.663) | (4.942) | (0.870) | (6.149) |
| Observations | 1225 | 1157 | 1034 | 943 | 1650 | 1558 |
| Adjusted R-squared | 0.444 | 0.646 | 0.055 | 0.400 | 0.234 | 0.597 |

Notes: Standard errors in parentheses; ***p < 0.001   **p < 0.01 * p< 0.05. Source: Our data came from four publicly available datasets: World Bank’s World Development Indicators; U.S. government’s foreignassistance.gov database; OECD Creditor Reporting System database; and the Institute of Health Metrics and Evaluation GBD Result’s Tool.

**Exhibit [G]. DID results for PEPFAR impact on male primary school disengagement rate by COP country income group (without India)**

| **Variables** | **Low-income COP countries**  **without India** | | **All COP countries**  **without India** | |
| --- | --- | --- | --- | --- |
|  | **Unadjusted** | **Adjusted** | **Unadjusted** | **Adjusted** |
| Time variable (=1 post-2004) | -3.010*** | -2.599*** | -3.010*** | -2.545*** |
|  | (0.793) | (0.733) | (0.853) | (0.753) |
| Intervention (=1 if PEPFAR) | 26.43*** | 8.140*** | 18.64*** | 6.363*** |
|  | (1.127) | (1.954) | (1.057) | (1.607) |
| Interaction term (PEPFAR impact estimate) | -18.59*** | -19.14*** | -12.59*** | -12.87*** |
|  | (1.502) | (1.316) | (1.403) | (1.189) |
| Country income group (=1 if middle income) |  | n/a |  | -2.321 |
|  |  | n/a |  | (1.226) |
| BL population |  | 3.50e-08* |  | -1.65e-08 |
|  |  | (1.52e-08) |  | (8.78e-09) |
| BL per capita non-PEPFAR donor spending on health (constant $) |  | 0.00301 |  | -0.000104 |
|  |  | (0.0198) |  | (0.0200) |
| BL per capita domestic health spending (current $) |  | -0.000801 |  | -0.00263 |
|  |  | (0.00226) |  | (0.00215) |
| BL GDP per capita, PPP (constant 2011 international $) |  | 0.000430*** |  | 0.000469*** |
|  |  | (0.0000812) |  | (0.0000811) |
| BL HIV prevalence (% of population ages 15-49) |  | -0.387*** |  | -0.355*** |
|  |  | (0.112) |  | (0.101) |
| BL life expectancy at birth |  | -0.609*** |  | -0.529*** |
|  |  | (0.109) |  | (0.110) |
| BL urban population (%) |  | -0.0461* |  | -0.0121 |
|  |  | (0.0229) |  | (0.0221) |
| BL school enrollment, secondary (% gross) |  | -0.140*** |  | -0.102*** |
|  |  | (0.0273) |  | (0.0251) |
| BL fertility rate (births per woman) |  | 1.484** |  | 1.499** |
|  |  | (0.491) |  | (0.475) |
| Recipient of US HIV aid before 2004 (=1 if yes) |  | 0.548 |  | 1.830 |
|  |  | (1.071) |  | (0.993) |
| Constant | 9.054*** | 57.28*** | 9.054*** | 49.94*** |
|  | (0.611) | (8.481) | (0.658) | (8.305) |
| Observations | 838 | 770 | 957 | 889 |
| Adjusted R-squared | 0.482 | 0.639 | 0.341 | 0.578 |

Notes: Standard errors in parentheses; ***p < 0.001   **p < 0.01 * p< 0.05. Source: Our data came from four publicly available datasets: World Bank’s World Development Indicators; U.S. government’s foreignassistance.gov database; OECD Creditor Reporting System database; and the Institute of Health Metrics and Evaluation GBD Result’s Tool.

**Exhibit [G]. DID results for PEPFAR impact on male primary school disengagement rate for PEPFAR countries by five-year time periods (without India and China)**

| **Variables** | **PEPFAR countries without India and China** | | | | | | |
| --- | --- | --- | --- | --- | --- | --- | --- |
|  | **Unadjusted** | | | | **Adjusted** | | |
|  | **2004-2008** | | **2009-2013** | **2014-2018** | **2004-2008** | **2009-2013** | **2014-2018** |
| Interaction term (PEPFAR impact estimate) | -7.189*** | -1.214 | | -0.396 | -5.567*** | -1.584* | -0.831* |
|  | (2.109) | (0.783) | | (0.376) | (1.391) | (0.680) | (0.346) |

**Exhibit [G]. DID results for PEPFAR impact on male primary school disengagement rate for COP countries by five-year time periods (without India)**

| **Variables** | **COP countries without India** | | | | | | |
| --- | --- | --- | --- | --- | --- | --- | --- |
|  | **Unadjusted** | | | | **Adjusted** | | |
|  | **2004-2008** | | **2009-2013** | **2014-2018** | **2004-2008** | **2009-2013** | **2014-2018** |
| Interaction term (PEPFAR impact estimate) | -9.740*** | -1.877* | | -0.971* | -9.233*** | -2.261* | -1.373** |
|  | (2.047) | (0.850) | | (0.391) | (1.636) | (0.976) | (0.472) |

**Exhibit H. Sensitivity test for PEPFAR impact on female employment rates**

- by PEPFAR/COP country income group
- by three five-year intervals of the post-treatment period for PEPFAR/COP country
- exclude China and India as PEPFAR/COP country

**Exhibit [H]. DID results for PEPFAR impact on female employment rate by PEPFAR country income group**

| **Variables** | **Low-income**  **PEPFAR countries** | | **Middle-income**  **PEPFAR countries** | | **All**  **PEPFAR countries** | |
| --- | --- | --- | --- | --- | --- | --- |
|  | **Unadjusted** | **Adjusted** | **Unadjusted** | **Adjusted** | **Unadjusted** | **Adjusted** |
| Time variable (=1 post-2004) | 2.484** | 2.869*** | 2.484*** | 2.869*** | 2.484** | 2.869*** |
|  | (0.868) | (0.800) | (0.737) | (0.685) | (0.858) | (0.805) |
| Intervention (=1 if PEPFAR) | 21.02*** | 4.707*** | 6.536*** | 4.275*** | 15.00*** | 2.084* |
|  | (0.898) | (1.220) | (0.837) | (0.939) | (0.790) | (0.885) |
| Interaction term (PEPFAR impact estimate) | -3.178** | -3.538** | -0.200 | -0.734 | -1.940 | -2.416* |
|  | (1.227) | (1.091) | (1.143) | (1.037) | (1.080) | (0.991) |
| Country income group (=1 if middle income) |  | n/a |  | n/a |  | -2.579** |
|  |  | n/a |  | n/a |  | (0.865) |
| BL population |  | -3.54e-08*** |  | 9.92e-09*** |  | -1.03e-08*** |
|  |  | (2.46e-09) |  | (1.83e-09) |  | (1.64e-09) |
| BL per capita non-PEPFAR donor spending on health (constant $) |  | 0.222*** |  | 0.483*** |  | 0.0579 |
|  |  | (0.0463) |  | (0.0465) |  | (0.0420) |
| BL per capita domestic health spending (current $) |  | 0.0235*** |  | 0.00348 |  | 0.0148*** |
|  |  | (0.00222) |  | (0.00187) |  | (0.00191) |
| BL GDP per capita, PPP (constant 2011 international $) |  | -0.000702*** |  | -0.000152* |  | -0.000435*** |
|  |  | (0.0000841) |  | (0.0000663) |  | (0.0000735) |
| BL HIV prevalence (% of population ages 15-49) |  | -0.720*** |  | -1.117*** |  | -1.116*** |
|  |  | (0.114) |  | (0.0975) |  | (0.0857) |
| BL life expectancy at birth |  | -1.205*** |  | -0.870*** |  | -1.149*** |
|  |  | (0.0891) |  | (0.0814) |  | (0.0737) |
| BL urban population (%) |  | -0.184*** |  | -0.0127 |  | -0.183*** |
|  |  | (0.0208) |  | (0.0188) |  | (0.0178) |
| BL school enrollment, secondary (% gross) |  | -0.182*** |  | -0.147*** |  | -0.184*** |
|  |  | (0.0216) |  | (0.0201) |  | (0.0186) |
| BL fertility rate (births per woman) |  | -4.997*** |  | -7.113*** |  | -5.152*** |
|  |  | (0.413) |  | (0.406) |  | (0.344) |
| Recipient of US HIV aid before 2004 (=1 if yes) |  | 6.505*** |  | 1.259 |  | 6.693*** |
|  |  | (0.726) |  | (0.840) |  | (0.595) |
| Constant | 35.64*** | 156.2*** | 35.64*** | 127.5*** | 35.64*** | 156.7*** |
|  | (0.635) | (7.101) | (0.540) | (6.370) | (0.628) | (5.680) |
| Observations | 2912 | 2660 | 2492 | 2184 | 3948 | 3612 |
| Adjusted R-squared | 0.256 | 0.424 | 0.054 | 0.221 | 0.147 | 0.334 |

Notes: Standard errors in parentheses; ***p < 0.001   **p < 0.01 * p< 0.05. Source: Our data came from four publicly available datasets: World Bank’s World Development Indicators; U.S. government’s foreignassistance.gov database; OECD Creditor Reporting System database; and the Institute of Health Metrics and Evaluation GBD Result’s Tool.

**Exhibit [H]. DID results for PEPFAR impact on female employment rate by COP country income group**

| **Variables** | **Low-income**  **COP countries** | | **Middle-income**  **COP countries** | | **All**  **COP countries** | |
| --- | --- | --- | --- | --- | --- | --- |
|  | **Unadjusted** | **Adjusted** | **Unadjusted** | **Adjusted** | **Unadjusted** | **Adjusted** |
| Time variable (=1 post-2004) | 2.484** | 2.869*** | 2.484** | 2.869*** | 2.484** | 2.869*** |
|  | (0.790) | (0.671) | (0.766) | (0.623) | (0.840) | (0.695) |
| Intervention (=1 if PEPFAR) | 28.88*** | 33.01*** | 2.709 | 13.59*** | 21.28*** | 15.88*** |
|  | (1.060) | (1.578) | (1.459) | (1.931) | (1.006) | (1.416) |
| Interaction term (PEPFAR impact estimate) | -4.520** | -4.908*** | 1.246 | 0.875 | -2.846* | -3.313** |
|  | (1.449) | (1.181) | (1.993) | (1.589) | (1.374) | (1.102) |
| Country income group (=1 if middle income) |  | n/a |  | n/a |  | -12.65*** |
|  |  | n/a |  | n/a |  | (1.022) |
| BL population |  | -4.14e-08*** |  | -6.17e-08*** |  | -3.73e-08*** |
|  |  | (2.21e-09) |  | (9.96e-09) |  | (2.22e-09) |
| BL per capita non-PEPFAR donor spending on health (constant $) |  | 0.714*** |  | 0.796*** |  | 0.523*** |
|  |  | (0.0490) |  | (0.0488) |  | (0.0484) |
| BL per capita domestic health spending (current $) |  | 0.0170*** |  | 0.0141*** |  | 0.0207*** |
|  |  | (0.00204) |  | (0.00202) |  | (0.00203) |
| BL GDP per capita, PPP (constant 2011 international $) |  | -0.000544*** |  | -0.000522*** |  | -0.000603*** |
|  |  | (0.0000740) |  | (0.0000710) |  | (0.0000759) |
| BL HIV prevalence (% of population ages 15-49) |  | -0.887*** |  | -1.546*** |  | -0.955*** |
|  |  | (0.111) |  | (0.103) |  | (0.0937) |
| BL life expectancy at birth |  | -0.339*** |  | -1.115*** |  | -0.345*** |
|  |  | (0.0974) |  | (0.0979) |  | (0.0959) |
| BL urban population (%) |  | 0.0132 |  | 0.150*** |  | 0.0327 |
|  |  | (0.0209) |  | (0.0207) |  | (0.0206) |
| BL school enrollment, secondary (% gross) |  | -0.174*** |  | -0.257*** |  | -0.240*** |
|  |  | (0.0226) |  | (0.0229) |  | (0.0224) |
| BL fertility rate (births per woman) |  | -4.002*** |  | -7.497*** |  | -3.346*** |
|  |  | (0.436) |  | (0.509) |  | (0.438) |
| Recipient of US HIV aid before 2004 (=1 if yes) |  | 0.326 |  | -9.101*** |  | 0.373 |
|  |  | (0.968) |  | (1.361) |  | (0.941) |
| Constant | 35.64*** | 79.97*** | 35.64*** | 144.8*** | 35.64*** | 93.83*** |
|  | (0.578) | (7.762) | (0.560) | (7.631) | (0.615) | (7.539) |
| Observations | 2072 | 1820 | 1708 | 1456 | 2324 | 2044 |
| Adjusted R-squared | 0.395 | 0.597 | 0.013 | 0.345 | 0.265 | 0.543 |

Notes: Standard errors in parentheses; ***p < 0.001   **p < 0.01 * p< 0.05. Source: Our data came from four publicly available datasets: World Bank’s World Development Indicators; U.S. government’s foreignassistance.gov database; OECD Creditor Reporting System database; and the Institute of Health Metrics and Evaluation GBD Result’s Tool.

**Exhibit [H]. DID results for PEPFAR impact on female employment rate for PEPFAR countries by five-year time periods**

| **Variables** | **PEPFAR countries** | | | | | | |
| --- | --- | --- | --- | --- | --- | --- | --- |
|  | **Unadjusted** | | | | **Adjusted** | | |
|  | **2004-2008** | | **2009-2013** | **2014-2018** | **2004-2008** | **2009-2013** | **2014-2018** |
| Interaction term (PEPFAR impact estimate) | -1.172 | -0.458 | | -0.309 | -1.492 | -0.529 | -0.395 |
|  | (1.515) | (0.877) | | (0.507) | (1.370) | (0.825) | (0.489) |

**Exhibit [H]. DID results for PEPFAR impact on female employment rate for COP countries by five-year time periods**

| **Variables** | **COP countries** | | | | | | |
| --- | --- | --- | --- | --- | --- | --- | --- |
|  | **Unadjusted** | | | | **Adjusted** | | |
|  | **2004-2008** | | **2009-2013** | **2014-2018** | **2004-2008** | **2009-2013** | **2014-2018** |
| Interaction term (PEPFAR impact estimate) | -1.852 | -0.613 | | -0.382 | -2.103 | -0.721 | -0.489 |
|  | (1.912) | (1.181) | | (0.665) | (1.483) | (0.921) | (0.546) |

**Exhibit [H]. DID results for PEPFAR impact on female employment rate by PEPFAR country income group (without India and China)**

| **Variables** | **Low-income PEPFAR countries without India** | | **Middle-income PEPFAR countries without China** | | **All PEPFAR countries without India and China** | |
| --- | --- | --- | --- | --- | --- | --- |
|  | **Unadjusted** | **Adjusted** | **Unadjusted** | **Adjusted** | **Unadjusted** | **Adjusted** |
| Time variable (=1 post-2004) | 2.484** | 2.869*** | 2.484*** | 2.869*** | 2.484** | 2.869*** |
|  | (0.858) | (0.782) | (0.730) | (0.684) | (0.855) | (0.781) |
| Intervention (=1 if PEPFAR) | 21.55*** | 5.416*** | 5.775*** | 3.864*** | 15.02*** | 1.834* |
|  | (0.893) | (1.196) | (0.836) | (0.942) | (0.791) | (0.862) |
| Interaction term (PEPFAR impact estimate) | -3.102* | -3.460** | 0.0877 | -0.424 | -1.782 | -2.253* |
|  | (1.220) | (1.072) | (1.142) | (1.045) | (1.080) | (0.967) |
| Country income group (=1 if middle income) |  | n/a |  | n/a |  | -4.234*** |
|  |  | n/a |  | n/a |  | (0.850) |
| BL population |  | -0.000000156*** |  | -2.84e-08*** |  | -7.04e-08*** |
|  |  | (1.01e-08) |  | (7.46e-09) |  | (6.70e-09) |
| BL per capita non-PEPFAR donor spending on health (constant $) |  | 0.0532 |  | 0.456*** |  | -0.0200 |
|  |  | (0.0473) |  | (0.0467) |  | (0.0416) |
| BL per capita domestic health spending (current $) |  | 0.0214*** |  | 0.00309 |  | 0.0144*** |
|  |  | (0.00218) |  | (0.00187) |  | (0.00186) |
| BL GDP per capita, PPP (constant 2011 international $) |  | -0.000696*** |  | -0.000139* |  | -0.000389*** |
|  |  | (0.0000822) |  | (0.0000662) |  | (0.0000715) |
| BL HIV prevalence (% of population ages 15-49) |  | -0.725*** |  | -1.175*** |  | -1.204*** |
|  |  | (0.111) |  | (0.0980) |  | (0.0836) |
| BL life expectancy at birth |  | -1.080*** |  | -0.903*** |  | -1.184*** |
|  |  | (0.0877) |  | (0.0815) |  | (0.0715) |
| BL urban population (%) |  | -0.178*** |  | -0.00434 |  | -0.175*** |
|  |  | (0.0204) |  | (0.0189) |  | (0.0173) |
| BL school enrollment, secondary (% gross) |  | -0.208*** |  | -0.142*** |  | -0.175*** |
|  |  | (0.0213) |  | (0.0201) |  | (0.0181) |
| BL fertility rate (births per woman) |  | -4.933*** |  | -7.176*** |  | -5.271*** |
|  |  | (0.404) |  | (0.406) |  | (0.334) |
| Recipient of US HIV aid before 2004 (=1 if yes) |  | 7.232*** |  | 2.591** |  | 8.014*** |
|  |  | (0.712) |  | (0.875) |  | (0.588) |
| Constant | 35.64*** | 152.3*** | 35.64*** | 129.9*** | 35.64*** | 160.7*** |
|  | (0.628) | (6.950) | (0.535) | (6.377) | (0.626) | (5.524) |
| Observations | 2884 | 2632 | 2464 | 2156 | 3892 | 3556 |
| Adjusted R-squared | 0.271 | 0.447 | 0.047 | 0.197 | 0.150 | 0.370 |

Notes: Standard errors in parentheses; ***p < 0.001   **p < 0.01 * p< 0.05. Source: Our data came from four publicly available datasets: World Bank’s World Development Indicators; U.S. government’s foreignassistance.gov database; OECD Creditor Reporting System database; and the Institute of Health Metrics and Evaluation GBD Result’s Tool.

**Exhibit [H]. DID results for PEPFAR impact on female employment rate by COP country income group (without India)**

| **Variables** | **Low-income COP countries**  **without India** | | **All COP countries**  **without India** | |
| --- | --- | --- | --- | --- |
|  | **Unadjusted** | **Adjusted** | **Unadjusted** | **Adjusted** |
| Time variable (=1 post-2004) | 2.484** | 2.869*** | 2.484** | 2.869*** |
|  | (0.762) | (0.644) | (0.827) | (0.693) |
| Intervention (=1 if PEPFAR) | 30.53*** | 35.05*** | 22.19*** | 16.74*** |
|  | (1.040) | (1.527) | (1.001) | (1.424) |
| Interaction term (PEPFAR impact estimate) | -4.399** | -4.780*** | -2.705* | -3.165** |
|  | (1.421) | (1.153) | (1.367) | (1.112) |
| Country income group (=1 if middle income) |  | n/a |  | -12.09*** |
|  |  | n/a |  | (1.024) |
| BL population |  | -0.000000222*** |  | -9.19e-08*** |
|  |  | (1.36e-08) |  | (9.57e-09) |
| BL per capita non-PEPFAR donor spending on health (constant $) |  | 0.550*** |  | 0.461*** |
|  |  | (0.0486) |  | (0.0495) |
| BL per capita domestic health spending (current $) |  | 0.0153*** |  | 0.0192*** |
|  |  | (0.00196) |  | (0.00204) |
| BL GDP per capita, PPP (constant 2011 international $) |  | -0.000573*** |  | -0.000585*** |
|  |  | (0.0000711) |  | (0.0000758) |
| BL HIV prevalence (% of population ages 15-49) |  | -1.214*** |  | -1.044*** |
|  |  | (0.110) |  | (0.0947) |
| BL life expectancy at birth |  | -0.369*** |  | -0.354*** |
|  |  | (0.0935) |  | (0.0957) |
| BL urban population (%) |  | 0.0448* |  | 0.0379 |
|  |  | (0.0202) |  | (0.0206) |
| BL school enrollment, secondary (% gross) |  | -0.128*** |  | -0.236*** |
|  |  | (0.0220) |  | (0.0223) |
| BL fertility rate (births per woman) |  | -3.309*** |  | -3.358*** |
|  |  | (0.422) |  | (0.437) |
| Recipient of US HIV aid before 2004 (=1 if yes) |  | 4.423*** |  | 1.541 |
|  |  | (0.978) |  | (0.960) |
| Constant | 35.64*** | 79.42*** | 35.64*** | 94.91*** |
|  | (0.558) | (7.450) | (0.605) | (7.525) |
| Observations | 2044 | 1792 | 2296 | 2016 |
| Adjusted R-squared | 0.438 | 0.628 | 0.289 | 0.544 |

Notes: Standard errors in parentheses; ***p < 0.001   **p < 0.01 * p< 0.05. Source: Our data came from four publicly available datasets: World Bank’s World Development Indicators; U.S. government’s foreignassistance.gov database; OECD Creditor Reporting System database; and the Institute of Health Metrics and Evaluation GBD Result’s Tool.

**Exhibit [H]. DID results for PEPFAR impact on female employment rate for PEPFAR countries by five-year time periods (without India and China)**

| **Variables** | **PEPFAR countries without India and China** | | | | | | |
| --- | --- | --- | --- | --- | --- | --- | --- |
|  | **Unadjusted** | | | | **Adjusted** | | |
|  | **2004-2008** | | **2009-2013** | **2014-2018** | **2004-2008** | **2009-2013** | **2014-2018** |
| Interaction term (PEPFAR impact estimate) | -1.088 | -0.412 | | -0.281 | -1.405 | -0.482 | -0.366 |
|  | (1.517) | (0.880) | | (0.508) | (1.331) | (0.812) | (0.482) |

**Exhibit [H]. DID results for PEPFAR impact on female employment rate for COP countries by five-year time periods (without India)**

| **Variables** | **COP countries without India** | | | | | | |
| --- | --- | --- | --- | --- | --- | --- | --- |
|  | **Unadjusted** | | | | **Adjusted** | | |
|  | **2004-2008** | | **2009-2013** | **2014-2018** | **2004-2008** | **2009-2013** | **2014-2018** |
| Interaction term (PEPFAR impact estimate) | -1.845 | -0.525 | | -0.336 | -2.093 | -0.629 | -0.442 |
|  | (1.909) | (1.164) | | (0.648) | (1.495) | (0.938) | (0.556) |

**Exhibit I. Sensitivity test for PEPFAR impact on male employment rates**

- by PEPFAR/COP country income group
- by three five-year intervals of the post-treatment period for PEPFAR/COP country
- exclude China and India as PEPFAR/COP country

**Exhibit [I]. DID results for PEPFAR impact on male employment rate by PEPFAR country income group**

| **Variables** | **Low-income**  **PEPFAR countries** | | **Middle-income**  **PEPFAR countries** | | **All**  **PEPFAR countries** | |
| --- | --- | --- | --- | --- | --- | --- |
|  | **Unadjusted** | **Adjusted** | **Unadjusted** | **Adjusted** | **Unadjusted** | **Adjusted** |
| Time variable (=1 post-2004) | -0.444 | -0.220 | -0.444 | -0.220 | -0.444 | -0.220 |
|  | (0.532) | (0.484) | (0.526) | (0.457) | (0.564) | (0.507) |
| Intervention (=1 if PEPFAR) | 7.439*** | 1.056 | 0.363 | -1.756** | 4.497*** | -0.557 |
|  | (0.551) | (0.738) | (0.597) | (0.627) | (0.520) | (0.557) |
| Interaction term (PEPFAR impact estimate) | -1.907* | -2.077** | -0.660 | -1.026 | -1.389 | -1.657** |
|  | (0.753) | (0.660) | (0.815) | (0.692) | (0.710) | (0.625) |
| Country income group (=1 if middle income) |  | n/a |  | n/a |  | -1.608** |
|  |  | n/a |  | n/a |  | (0.545) |
| BL population |  | 7.30e-09*** |  | 5.23e-09*** |  | 4.36e-09*** |
|  |  | (1.49e-09) |  | (1.22e-09) |  | (1.03e-09) |
| BL per capita non-PEPFAR donor spending on health (constant $) |  | -0.142*** |  | -0.0442 |  | -0.227*** |
|  |  | (0.0280) |  | (0.0310) |  | (0.0264) |
| BL per capita domestic health spending (current $) |  | -0.00882*** |  | -0.0132*** |  | -0.00976*** |
|  |  | (0.00134) |  | (0.00125) |  | (0.00121) |
| BL GDP per capita, PPP (constant 2011 international $) |  | 0.000154** |  | 0.000331*** |  | 0.000208*** |
|  |  | (0.0000509) |  | (0.0000442) |  | (0.0000463) |
| BL HIV prevalence (% of population ages 15-49) |  | 0.676*** |  | 0.331*** |  | 0.333*** |
|  |  | (0.0688) |  | (0.0651) |  | (0.0540) |
| BL life expectancy at birth |  | 0.921*** |  | 1.033*** |  | 0.826*** |
|  |  | (0.0539) |  | (0.0543) |  | (0.0464) |
| BL urban population (%) |  | -0.0585*** |  | 0.0126 |  | -0.0392*** |
|  |  | (0.0126) |  | (0.0125) |  | (0.0112) |
| BL school enrollment, secondary (% gross) |  | -0.213*** |  | -0.0977*** |  | -0.213*** |
|  |  | (0.0131) |  | (0.0134) |  | (0.0117) |
| BL fertility rate (births per woman) |  | 1.483*** |  | 2.321*** |  | 1.224*** |
|  |  | (0.250) |  | (0.271) |  | (0.217) |
| Recipient of US HIV aid before 2004 (=1 if yes) |  | 2.026*** |  | 5.420*** |  | 3.739*** |
|  |  | (0.439) |  | (0.560) |  | (0.375) |
| Constant | 66.72*** | 20.97*** | 66.72*** | -2.580 | 66.72*** | 28.96*** |
|  | (0.390) | (4.294) | (0.385) | (4.250) | (0.413) | (3.578) |
| Observations | 2912 | 2660 | 2492 | 2184 | 3948 | 3612 |
| Adjusted R-squared | 0.096 | 0.323 | 0.000 | 0.312 | 0.031 | 0.314 |

Notes: Standard errors in parentheses; ***p < 0.001   **p < 0.01 * p< 0.05. Source: Our data came from four publicly available datasets: World Bank’s World Development Indicators; U.S. government’s foreignassistance.gov database; OECD Creditor Reporting System database; and the Institute of Health Metrics and Evaluation GBD Result’s Tool.

**Exhibit [I]. DID results for PEPFAR impact on male employment rate by COP country income group**

| **Variables** | **Low-income**  **COP countries** | | **Middle-income**  **COP countries** | | **All**  **COP countries** | |
| --- | --- | --- | --- | --- | --- | --- |
|  | **Unadjusted** | **Adjusted** | **Unadjusted** | **Adjusted** | **Unadjusted** | **Adjusted** |
| Time variable (=1 post-2004) | -0.444 | -0.220 | -0.444 | -0.220 | -0.444 | -0.220 |
|  | (0.481) | (0.405) | (0.486) | (0.335) | (0.523) | (0.406) |
| Intervention (=1 if PEPFAR) | 9.664*** | 11.40*** | -4.178*** | 6.420*** | 5.645*** | 5.338*** |
|  | (0.646) | (0.952) | (0.925) | (1.039) | (0.627) | (0.827) |
| Interaction term (PEPFAR impact estimate) | -2.087* | -2.189** | 0.106 | -0.236 | -1.451 | -1.650* |
|  | (0.882) | (0.713) | (1.264) | (0.855) | (0.856) | (0.644) |
| Country income group (=1 if middle income) |  | n/a |  | n/a |  | -5.316*** |
|  |  | n/a |  | n/a |  | (0.597) |
| BL population |  | 1.31e-09 |  | 1.74e-08** |  | 2.05e-09 |
|  |  | (1.33e-09) |  | (5.36e-09) |  | (1.30e-09) |
| BL per capita non-PEPFAR donor spending on health (constant $) |  | 0.0647* |  | 0.116*** |  | 0.0180 |
|  |  | (0.0296) |  | (0.0262) |  | (0.0283) |
| BL per capita domestic health spending (current $) |  | -0.0139*** |  | -0.0128*** |  | -0.0136*** |
|  |  | (0.00123) |  | (0.00109) |  | (0.00119) |
| BL GDP per capita, PPP (constant 2011 international $) |  | 0.000268*** |  | 0.000269*** |  | 0.000294*** |
|  |  | (0.0000446) |  | (0.0000382) |  | (0.0000443) |
| BL HIV prevalence (% of population ages 15-49) |  | 0.664*** |  | 0.404*** |  | 0.517*** |
|  |  | (0.0671) |  | (0.0555) |  | (0.0548) |
| BL life expectancy at birth |  | 1.203*** |  | 1.350*** |  | 1.246*** |
|  |  | (0.0588) |  | (0.0527) |  | (0.0560) |
| BL urban population (%) |  | 0.0258* |  | 0.0219 |  | 0.0196 |
|  |  | (0.0126) |  | (0.0112) |  | (0.0120) |
| BL school enrollment, secondary (% gross) |  | -0.120*** |  | -0.0786*** |  | -0.146*** |
|  |  | (0.0136) |  | (0.0123) |  | (0.0131) |
| BL fertility rate (births per woman) |  | 2.058*** |  | 3.613*** |  | 2.303*** |
|  |  | (0.263) |  | (0.274) |  | (0.256) |
| Recipient of US HIV aid before 2004 (=1 if yes) |  | 2.343*** |  | -1.033 |  | 2.447*** |
|  |  | (0.584) |  | (0.733) |  | (0.550) |
| Constant | 66.72*** | -12.22** | 66.72*** | -30.47*** | 66.72*** | -8.904* |
|  | (0.352) | (4.683) | (0.355) | (4.106) | (0.383) | (4.405) |
| Observations | 2072 | 1820 | 1708 | 1456 | 2324 | 2044 |
| Adjusted R-squared | 0.157 | 0.425 | 0.023 | 0.522 | 0.055 | 0.460 |

Notes: Standard errors in parentheses; ***p < 0.001   **p < 0.01 * p< 0.05. Source: Our data came from four publicly available datasets: World Bank’s World Development Indicators; U.S. government’s foreignassistance.gov database; OECD Creditor Reporting System database; and the Institute of Health Metrics and Evaluation GBD Result’s Tool.

**Exhibit [I]. DID results for PEPFAR impact on male employment rate for PEPFAR countries by five-year time periods**

| **Variables** | **PEPFAR countries** | | | | | | |
| --- | --- | --- | --- | --- | --- | --- | --- |
|  | **Unadjusted** | | | | **Adjusted** | | |
|  | **2004-2008** | | **2009-2013** | **2014-2018** | **2004-2008** | **2009-2013** | **2014-2018** |
| Interaction term (PEPFAR impact estimate) | -0.864 | -0.179 | | -0.346 | -1.101 | -0.186 | -0.370 |
|  | (0.981) | (0.566) | | (0.318) | (0.838) | (0.491) | (0.288) |

**Exhibit [i]. DID results for PEPFAR impact on male employment rate for COP countries by five-year time periods**

| **Variables** | **COP countries** | | | | | | |
| --- | --- | --- | --- | --- | --- | --- | --- |
|  | **Unadjusted** | | | | **Adjusted** | | |
|  | **2004-2008** | | **2009-2013** | **2014-2018** | **2004-2008** | **2009-2013** | **2014-2018** |
| Interaction term (PEPFAR impact estimate) | -0.840 | -0.232 | | -0.378 | -0.964 | -0.263 | -0.423 |
|  | (1.177) | (0.782) | | (0.445) | (0.837) | (0.593) | (0.359) |

**Exhibit [I]. DID results for PEPFAR impact on male employment rate by PEPFAR country income group (without India and China)**

| **Variables** | **Low-income PEPFAR countries without India** | | **Middle-income PEPFAR countries without China** | | **All PEPFAR countries without India and China** | |
| --- | --- | --- | --- | --- | --- | --- |
|  | **Unadjusted** | **Adjusted** | **Unadjusted** | **Adjusted** | **Unadjusted** | **Adjusted** |
| Time variable (=1 post-2004) | -0.444 | -0.220 | -0.444 | -0.220 | -0.444 | -0.220 |
|  | (0.534) | (0.485) | (0.525) | (0.454) | (0.566) | (0.506) |
| Intervention (=1 if PEPFAR) | 7.293*** | 0.901 | -0.0153 | -1.632** | 4.269*** | -0.775 |
|  | (0.555) | (0.741) | (0.601) | (0.626) | (0.523) | (0.558) |
| Interaction term (PEPFAR impact estimate) | -1.897* | -2.066** | -0.504 | -0.860 | -1.321 | -1.587* |
|  | (0.758) | (0.665) | (0.821) | (0.694) | (0.715) | (0.626) |
| Country income group (=1 if middle income) |  | n/a |  | n/a |  | -1.555** |
|  |  | n/a |  | n/a |  | (0.550) |
| BL population |  | 3.12e-08*** |  | 3.84e-08*** |  | 3.88e-08*** |
|  |  | (6.29e-09) |  | (4.96e-09) |  | (4.34e-09) |
| BL per capita non-PEPFAR donor spending on health (constant $) |  | -0.108*** |  | -0.0206 |  | -0.181*** |
|  |  | (0.0293) |  | (0.0310) |  | (0.0270) |
| BL per capita domestic health spending (current $) |  | -0.00840*** |  | -0.0128*** |  | -0.00918*** |
|  |  | (0.00135) |  | (0.00124) |  | (0.00121) |
| BL GDP per capita, PPP (constant 2011 international $) |  | 0.000153** |  | 0.000320*** |  | 0.000193*** |
|  |  | (0.0000510) |  | (0.0000440) |  | (0.0000463) |
| BL HIV prevalence (% of population ages 15-49) |  | 0.677*** |  | 0.382*** |  | 0.372*** |
|  |  | (0.0689) |  | (0.0651) |  | (0.0541) |
| BL life expectancy at birth |  | 0.896*** |  | 1.062*** |  | 0.836*** |
|  |  | (0.0543) |  | (0.0542) |  | (0.0463) |
| BL urban population (%) |  | -0.0597*** |  | 0.00539 |  | -0.0444*** |
|  |  | (0.0126) |  | (0.0125) |  | (0.0112) |
| BL school enrollment, secondary (% gross) |  | -0.208*** |  | -0.102*** |  | -0.210*** |
|  |  | (0.0132) |  | (0.0134) |  | (0.0117) |
| BL fertility rate (births per woman) |  | 1.470*** |  | 2.377*** |  | 1.320*** |
|  |  | (0.250) |  | (0.270) |  | (0.217) |
| Recipient of US HIV aid before 2004 (=1 if yes) |  | 1.882*** |  | 4.267*** |  | 3.170*** |
|  |  | (0.442) |  | (0.582) |  | (0.381) |
| Constant | 66.72*** | 21.76*** | 66.72*** | -4.602 | 66.72*** | 27.26*** |
|  | (0.391) | (4.309) | (0.384) | (4.237) | (0.414) | (3.576) |
| Observations | 2884 | 2632 | 2464 | 2156 | 3892 | 3556 |
| Adjusted R-squared | 0.092 | 0.321 | 0.000 | 0.317 | 0.028 | 0.319 |

Notes: Standard errors in parentheses; ***p < 0.001   **p < 0.01 * p< 0.05. Source: Our data came from four publicly available datasets: World Bank’s World Development Indicators; U.S. government’s foreignassistance.gov database; OECD Creditor Reporting System database; and the Institute of Health Metrics and Evaluation GBD Result’s Tool.

**Exhibit [i]. DID results for PEPFAR impact on male employment rate by COP country income group (without India)**

| **Variables** | **Low-income COP countries**  **without India** | | **All COP countries**  **without India** | |
| --- | --- | --- | --- | --- |
|  | **Unadjusted** | **Adjusted** | **Unadjusted** | **Adjusted** |
| Time variable (=1 post-2004) | -0.444 | -0.220 | -0.444 | -0.220 |
|  | (0.483) | (0.405) | (0.524) | (0.408) |
| Intervention (=1 if PEPFAR) | 9.416*** | 11.87*** | 5.338*** | 5.450*** |
|  | (0.659) | (0.961) | (0.634) | (0.839) |
| Interaction term (PEPFAR impact estimate) | -2.073* | -2.167** | -1.419 | -1.616* |
|  | (0.901) | (0.725) | (0.866) | (0.655) |
| Country income group (=1 if middle income) |  | n/a |  | -5.238*** |
|  |  | n/a |  | (0.603) |
| BL population |  | -4.03e-08*** |  | -5.50e-09 |
|  |  | (8.55e-09) |  | (5.64e-09) |
| BL per capita non-PEPFAR donor spending on health (constant $) |  | 0.0270 |  | 0.00934 |
|  |  | (0.0306) |  | (0.0292) |
| BL per capita domestic health spending (current $) |  | -0.0143*** |  | -0.0138*** |
|  |  | (0.00124) |  | (0.00120) |
| BL GDP per capita, PPP (constant 2011 international $) |  | 0.000262*** |  | 0.000296*** |
|  |  | (0.0000447) |  | (0.0000446) |
| BL HIV prevalence (% of population ages 15-49) |  | 0.588*** |  | 0.505*** |
|  |  | (0.0689) |  | (0.0558) |
| BL life expectancy at birth |  | 1.196*** |  | 1.245*** |
|  |  | (0.0588) |  | (0.0564) |
| BL urban population (%) |  | 0.0330** |  | 0.0203 |
|  |  | (0.0127) |  | (0.0121) |
| BL school enrollment, secondary (% gross) |  | -0.109*** |  | -0.146*** |
|  |  | (0.0138) |  | (0.0132) |
| BL fertility rate (births per woman) |  | 2.218*** |  | 2.302*** |
|  |  | (0.265) |  | (0.258) |
| Recipient of US HIV aid before 2004 (=1 if yes) |  | 3.288*** |  | 2.608*** |
|  |  | (0.615) |  | (0.566) |
| Constant | 66.72*** | -12.34** | 66.72*** | -8.755* |
|  | (0.354) | (4.687) | (0.383) | (4.434) |
| Observations | 2044 | 1792 | 2296 | 2016 |
| Adjusted R-squared | 0.147 | 0.422 | 0.049 | 0.450 |

Notes: Standard errors in parentheses; ***p < 0.001   **p < 0.01 * p< 0.05. Source: Our data came from four publicly available datasets: World Bank’s World Development Indicators; U.S. government’s foreignassistance.gov database; OECD Creditor Reporting System database; and the Institute of Health Metrics and Evaluation GBD Result’s Tool.

**Exhibit [I]. DID results for PEPFAR impact on male employment rate for PEPFAR countries by five-year time periods (without India and China)**

| **Variables** | **PEPFAR countries without India and China** | | | | | | |
| --- | --- | --- | --- | --- | --- | --- | --- |
|  | **Unadjusted** | | | | **Adjusted** | | |
|  | **2004-2008** | | **2009-2013** | **2014-2018** | **2004-2008** | **2009-2013** | **2014-2018** |
| Interaction term (PEPFAR impact estimate) | -0.823 | -0.164 | | -0.334 | -1.058 | -0.172 | -0.357 |
|  | (0.987) | (0.571) | | (0.321) | (0.839) | (0.493) | (0.290) |

**Exhibit [i]. DID results for PEPFAR impact on male employment rate for COP countries by five-year time periods (without India)**

| **Variables** | **COP countries without India** | | | | | | |
| --- | --- | --- | --- | --- | --- | --- | --- |
|  | **Unadjusted** | | | | **Adjusted** | | |
|  | **2004-2008** | | **2009-2013** | **2014-2018** | **2004-2008** | **2009-2013** | **2014-2018** |
| Interaction term (PEPFAR impact estimate) | -0.853 | -0.208 | | -0.358 | -0.973 | -0.240 | -0.403 |
|  | (1.189) | (0.796) | | (0.453) | (0.852) | (0.608) | (0.369) |
