# Supplementary material for: Analysis of economic and educational spillover effects in PEPFAR countries
Source: PLoS One. 2023 Dec 29;18(12):e0289909. doi: 10.1371/journal.pone.0289909 (PMC10756543; doi:10.1371/journal.pone.0289909)
Supplement: Attachment — Submitted filename: RESPONSE TO REVIEWERS.docx [file pone.0289909.s002.docx]

RESPONSE TO REVIEWERS FOR PONE-D-23-22009

**Analysis of Economic and Educational Spillover Effects in PEPFAR Countries**

**Editor Comments**

Please include the following items when submitting your revised manuscript:

A rebuttal letter that responds to each point raised by the academic editor and reviewer(s). You should upload this letter as a separate file labeled 'Response to Reviewers'.

A marked-up copy of your manuscript that highlights changes made to the original version. You should upload this as a separate file labeled 'Revised Manuscript with Track Changes'.

An unmarked version of your revised paper without tracked changes. You should upload this as a separate file labeled 'Manuscript'.

**We have prepared a response to each point raised by the academic editor and reviewer (i.e., this document), along with a revised version of the manuscript with and without track changes.**

Journal Requirements:

When submitting your revision, we need you to address these additional requirements.

1. Please ensure that your manuscript meets PLOS ONE's style requirements, including those for file naming. The PLOS ONEstyle templates can be found at

<https://journals.plos.org/plosone/s/file?id=wjVg/PLOSOne_formatting_sample_main_body.pdf> and 
<https://journals.plos.org/plosone/s/file?id=ba62/PLOSOne_formatting_sample_title_authors_affiliations.pdf>

Thank you. Completed.

2. In your Data Availability statement, you have not specified where the minimal data set underlying the results described in your manuscript can be found. PLOS defines a study's minimal data set as the underlying data used to reach the conclusions drawn in the manuscript and any additional data required to replicate the reported study findings in their entirety. All PLOS journals require that the minimal data set be made fully available. For more information about our data policy, please see <http://journals.plos.org/plosone/s/data-availability>.

Upon re-submitting your revised manuscript, please upload your study’s minimal underlying data set as either Supporting Information files or to a stable, public repository and include the relevant URLs, DOIs, or accession numbers within your revised cover letter. For a list of acceptable repositories, please see <http://journals.plos.org/plosone/s/data-availability#loc-recommended-repositories>. Any potentially identifying patient information must be fully anonymized.

Important: If there are ethical or legal restrictions to sharing your data publicly, please explain these restrictions in detail. Please see our guidelines for more information on what we consider unacceptable restrictions to publicly sharing data: <http://journals.plos.org/plosone/s/data-availability#loc-unacceptable-data-access-restrictions>. Note that it is not acceptable for the authors to be the sole named individuals responsible for ensuring data access.

We will update your Data Availability statement to reflect the information you provide in your cover letter.

Our data came from four publicly available datasets: World Bank’s World Development Indicators (www.databank.worldbank.org); U.S. government’s foreign assistance.gov database (www.ForeignAssistance.gov); OECD Creditor Reporting System database (www.stats.oecd.org); and the Institute of Health Metrics and Evaluation GBD Results Tool (www.healthdata.org).

3. We note that you have stated that you will provide repository information for your data at acceptance. Should your manuscript be accepted for publication, we will hold it until you provide the relevant accession numbers or DOIs necessary to access your data. If you wish to make changes to your Data Availability statement, please describe these changes in your cover letter and we will update your Data Availability statement to reflect the information you provide.

Thank you.

4. Please include captions for your Supporting Information files at the end of your manuscript, and update any in-text citations to match accordingly. Please see our Supporting Information guidelines for more information: <http://journals.plos.org/plosone/s/supporting-information>.

Completed.

5. Please review your reference list to ensure that it is complete and correct. If you have cited papers that have been retracted, please include the rationale for doing so in the manuscript text, or remove these references and replace them with relevant current references. Any changes to the reference list should be mentioned in the rebuttal letter that accompanies your revised manuscript. If you need to cite a retracted article, indicate the article’s retracted status in the References list and also include a citation and full reference for the retraction notice.

Completed.

**Additional Editor Comments:**

The manuscript is well written and clearly illustrated the impact of PEPFAR beyond the primary objectives of the initiative over the years.  Please review references, tables and figures to ensure that they are all aligned with PLOS ONE’s publication criteria.

Thank you. Completed.

**Additional Editor Comments:**

The manuscript is well written and clearly illustrated the impact of PEPFAR beyond the primary objectives of the initiative over the years.  Please review references, tables and figures to ensure that they are all aligned with PLOS ONE’s publication criteria.

Thank you. Completed.

Reviewers' comments:

**Comments to the Author**

1. Is the manuscript technically sound, and do the data support the conclusions?

The manuscript must describe a technically sound piece of scientific research with data that supports the conclusions. Experiments must have been conducted rigorously, with appropriate controls, replication, and sample sizes. The conclusions must be drawn appropriately based on the data presented.

Reviewer #1: Yes

2. Has the statistical analysis been performed appropriately and rigorously?

Reviewer #1: I Don't Know

3. Have the authors made all data underlying the findings in their manuscript fully available?

The [PLOS Data policy](http://www.plosone.org/static/policies.action#sharing) requires authors to make all data underlying the findings described in their manuscript fully available without restriction, with rare exception (please refer to the Data Availability Statement in the manuscript PDF file). The data should be provided as part of the manuscript or its supporting information, or deposited to a public repository. For example, in addition to summary statistics, the data points behind means, medians and variance measures should be available. If there are restrictions on publicly sharing data—e.g. participant privacy or use of data from a third party—those must be specified.

Reviewer #1: Yes

4. Is the manuscript presented in an intelligible fashion and written in standard English?

PLOS ONE does not copyedit accepted manuscripts, so the language in submitted articles must be clear, correct, and unambiguous. Any typographical or grammatical errors should be corrected at revision, so please note any specific errors here.

Reviewer #1: Yes

5. Review Comments to the Author

Please use the space provided to explain your answers to the questions above. You may also include additional comments for the author, including concerns about dual publication, research ethics, or publication ethics. (Please upload your review as an attachment if it exceeds 20,000 characters)

Reviewer #1: The manuscript is technically sound and has provided in-depth analysis and impact of PEPFAR beyond health. The contents may serve as great advocacy tool for continuous funding. Another area that this paper should have added to this analysis is OVC (orphans and vulnerable children) program. PEPFAR has substantially invested in OVC programs beyond what have been invested in DREAMS. This paper discuss DREAMS but not OVC. I recommend exploring how billions of dollars invested in OVC programs have impacted on some of the analysis provided in this paper.

Thank you for this comment. We have added a discussion of OVC to that of DREAMS to provide context for the possible programs within PEPFAR that may have led to the observed educational impacts on girls and boys in secondary school.
